# Mincle-dependent Th17 adjuvanticity requires TNFR1 signaling in myeloid cells

**DOI:** 10.3389/fimmu.2026.1746618

**Published:** 2026-02-09

**Authors:** Robert Blamberg, Carl Haberkamp, Gabriel Kristian Pedersen, Ida Rosenkrands, Ulrike Schleicher, Barbara U. Schraml, Sho Yamasaki, Roland Lang

**Affiliations:** 1 Institute of Clinical Microbiology, Immunology and Hygiene, Universitätsklinikum Erlangen, Friedrich-Alexander-Universität Erlangen-Nürnberg, Erlangen, Germany; 2 Center for Vaccine Research, Statens Serum Institut, Copenhagen, Denmark; 3 FAU Profile Center Immunomedicine (FAU I-MED), Friedrich-Alexander-Universität (FAU), Erlangen-Nürnberg, Erlangen, Germany; 4 Biomedical Center Munich, Institute of Immunology, Faculty of Medicine, Ludwig-Maximilians-Universität München, Planegg-Martinsried, Germany; 5 Department of Molecular Immunology, Research Institute for Microbial Diseases (RIMD), Osaka University, Suita, Japan

**Keywords:** CAF01, Mincle, TDB, TDM, Th17, TNF, TNFR1, vaccination

## Abstract

Successful induction of protective T cells by recombinant protein vaccines requires adjuvants. The liposomal adjuvant system CAF01 induces robust Th17 responses in mice. CAF01 contains the synthetic glycolipid trehalose-6,6-dibehenate (TDB), whose recognition by the C-type lectin receptor Mincle is required for Th17 induction. In previous work, we identified a pivotal role of TNF in upregulation of Mincle expression in macrophages and Th17 adjuvanticity of CAF01. The question has remained on which cell type(s) TNF acts to mediate the Th17 adjuvanticity of CAF01, and whether TNF-induced Mincle upregulation is causative. We used conditional TNFR1-deficient mice to dissect cell type-specific contributions of TNF signaling in myeloid cells, DC and T cells to Th17 induction by the recombinant tuberculosis fusion protein H1 adjuvanted with CAF01. LysM-Cre-mediated deletion of TNFR1 on myeloid cells completely abrogated vaccine-induced Th17 differentiation, replicating the phenotype in mice deficient in TNF or treated with the TNF blocker Etanercept. In contrast, TNFR1 deletion in DC by Clec9a-Cre did not affect Th17 induction, and by CD11c-Cre only partially reduced Th17 cells. T cell-specific deletion of TNFR1 by Lck-Cre had no impact on Th17 differentiation after vaccination. TNFR1 was expressed highly, and deleted efficiently via LysM-Cre, in monocytes and in neutrophils. We recently showed that neutrophils are not required for the adjuvant effect of CAF01, but monocytes are essential. Therefore, we analyzed activation of monocytes by TDB and observed robust upregulation of Mincle expression and of the Th17-inducing cytokines IL-1β and IL-6, that was inhibited by Etanercept. Finally, we asked whether Th17 induction by TNF is causally linked to Mincle upregulation. Constitutive, TNF-independent transgenic Mincle expression partially restored Th17 induction by CAF01 despite TNF blockade. Thus, upregulation of Mincle by TNF plays a causal role, likely by enabling production of Th17-polarizing cytokines by myeloid cells upon enhanced sensing of the adjuvant component TDB.

## Introduction

TNF is a key proinflammatory cytokine that contributes to various protective immune functions. It can signal via two TNF receptors: TNFR1, which is associated with pro-inflammatory functions, and TNFR2, which is involved in tissue regeneration and cell survival (1). Besides its beneficial effects in host defense against infection, TNF also plays a crucial negative role in many chronic inflammatory diseases like rheumatoid arthritis (RA), seronegative spondyloarthropathies, and inflammatory bowel disease (IBD), where a standard treatment strategy involves TNF blockade by antibodies (e.g. Infliximab or Adalimumab) or the human TNFR2-Fc fusion protein Etanercept (2). Unwanted side effects of TNF blockade include increased susceptibility to fungal and bacterial infections, including reactivation of latent tuberculosis (3). In addition, several studies reported an impaired immune response to vaccination due to TNF blockade, including reduced antibody responses to T cell-dependent vaccines for influenza (4–6) and hepatitis B (7–9) as well as for T cell-independent pneumococcal polysaccharide vaccines (9, 10).

Subunit vaccines consist of highly purified, often recombinant, antigens that can be processed and presented by antigen-presenting cells (APC) to T cells (signal 1). Their immunogenicity for T cells depends on adjuvants that increase costimulatory molecules expression (signal 2) and production of cytokines to promote and modulate Th cell differentiation (signal 3) (11, 12). Aluminum salts have been used as adjuvants in humans for nearly a century to induce antibody responses, but fail to generate strong T cell responses that are required to protect from intracellular pathogens (13). Complete Freund’s adjuvant (CFA), an emulsion of heat-killed *Mycobacterium tuberculosis* (MTB) in mineral oil, has been used in experimental animals for many decades to induce strong cellular immune responses, including T helper (Th)1 and Th17 cells, but its inflammatory side effects preclude its use in humans (14).

Inducing pathogen-specific Th17, along with Th1 cells, can be beneficial for protection against various intra- and extracellular bacteria and fungi. For example, to clear the extracellular bacterium *Klebsiella pneumoniae* in mice, IL-17 is essential to induce neutrophil recruitment (15). IL-17 activates macrophage killing of *Bordetella pertussis* and plays a role in the successful vaccination with whole-cell pertussis vaccines (16). Besides the recruitment of innate immune cells and the induction of other pro-inflammatory molecules, IL-17 promotes Th1 immunity. For example, it is critical to induce a Th1 response in *Chlamydia muridarum-*infected mice (17), and in humans deficient in functional RORγt, the Th17 lineage-defining transcription factor (18), an impaired IFNγ response to mycobacteria was observed (19). Further, induction of Th17 cells by the only licensed vaccine for tuberculosis, *Mycobacterium bovis* Bacille Calmette-Guérin (BCG), is required for protection after infection by recruiting Th1 cells (20) (21).

Research on innate immune recognition of infectious danger signals through pattern recognition receptors (PRR) has led to the development of new, molecularly defined adjuvants that induce and shape both cellular and humoral immune responses (22). One new adjuvant system named CAF01 (Cationic Adjuvant Formulation 01) induces a distinctive, mixed Th1/Th17 response, setting it apart from other next-generation adjuvants (13). CAF01 consists of cationic N,N-dimethyl-N,N-dioctadecylammonium (DDA) liposomes, whose membrane contains the glycolipid trehalose-6,6’-dibehenate (TDB) (23). TDB is a synthetic, less toxic analog of trehalose-6,6’-dimycolate (TDM), an abundant and immunostimulatory mycobacterial cell wall glycolipid (also known as cord factor). TDM and TDB bind to the activating PRR Mincle (macrophage-inducible C-type lectin) (24, 25). The Th17-inducing ability of CAF01 depends on the recognition of its component TDB by Mincle (25) and the resulting activation of the Syk-Card9 signaling pathway (26). Consistent with the abundance of TDM in the mycobacterial cell wall, the Th17-inducing effect of Complete Freund’s adjuvant is strongly reduced in Mincle- and abrogated in Card9-deficient mice (27).

Mincle is expressed on myeloid cells, such as monocytes, macrophages and neutrophils, as well as on some dendritic cells (DC) (28). Mincle is generally not expressed on adaptive immune cells, but some reports have found it under special circumstances on B cells (29, 30) and T cells (31). Mincle expression is low in resting macrophages, but upregulated after stimulation with LPS (32) or the Mincle-ligands TDB and TDM, leading to enhanced activation and pro-inflammatory signaling (33). We previously showed that TDB-/TDM-induced production of TNF was sufficient and essential for Mincle upregulation in macrophages, which was dependent on signaling via TNFR1 (34). The function of TNF in the induced expression of Mincle is conserved in human monocyte-derived macrophages after stimulation with BCG or LPS (35). Upon vaccination in mice, genetic deletion of TNF or pharmacological blockade by Etanercept prevents CAF01-induced Th17 induction, but does not affect the IFNγ response (34).

While these findings established the essential function of TNF in CAF01-adjuvanted immunization, the cell type on which it acts to enable Th17 differentiation has remained undefined. TNF receptors are expressed broadly in different tissues and immune cell types. We hypothesized that TNFR1 may be essential for Th17 induction in our vaccination model because of its pro-inflammatory function and its requirement for Mincle upregulation in macrophages. TNF-induced Mincle expression in macrophages, monocytes or in DC could enhance the sensing of the adjuvant TDB, leading to stronger upregulation of MHC-II (enhanced signal 1) or of co-stimulatory molecules on APC (enhanced signal 2). Alternatively, it could increase the production of cytokines, driving Th17 differentiation or maintenance (enhanced signal 3). Such cytokines include IL-6, IL-1, TGF-β, and IL-23 (36–40). Another possibility is that TNF may promote Th17 differentiation through TNFR1 signaling in CD4+ T cells. Conditional TNFR1 knockout mice crossed with cell-type-specific Cre-deleter strains provide a genetically defined system to dissect these pleiotropic effects of TNF (41). These mice have been employed to elucidate that for protection against MTB infection, TNFR1 signaling in T cells is dispensable but essential in myeloid cells (42). Here, we aimed to determine which immune cell type depends on TNF signaling to elicit both humoral and cellular immune responses following immunization with the recombinant MTB fusion protein H1 (43) adjuvanted with CAF01. Hence, conditional TNFR1 knockout mice were generated using Cre-deleter strains specific for myeloid cells, dendritic cells (DC), or T cells. Furthermore, we employed Mincle-transgenic mice to investigate whether impaired vaccine responses during TNF blockade can be restored by re-establishing robust Mincle expression.

Our results show that TNFR1 on myeloid cells is essential for inducing a Th17 response by H1/CAF01. TNFR1 on DC appears to contribute but is not essential, while TNFR1 on T cells is dispensable. Together, in the H1/CAF01 model, the lack of Mincle upregulation, probably on myeloid cells, can explain most of the effects of the TNF blocker.

## Methods

### Mice

All mice were bred and housed under specific pathogen-free conditions at the Präklinische Experimentelle Tierzentrum of the Medical Faculty in Erlangen, Germany, or at the Statens Serum Institute, Denmark. Frozen sperm cells of TNFR1^fl/wt^ mice (41) were obtained from the European Mouse Mutant Archive in Athens, Greece (EMMA strain 11019, B6.129P2(Cg)-Tnfrsf1a^tm3.3Gkl/Flmg^, archived on a C57BL/6 background), and used to re-derive the mice by *in vitro* fertilization at the Transgenic Facility of the Friedrich-Alexander-University Erlangen-Nürnberg. To generate cell type-specific conditional TNFR1 KO lines, TNFR1^fl/fl^ mice were crossed with LysM-Cre (B6.Lyz2^tm1(cre)Ifo^ (44);), Clec9a-Cre B6J.B6N(Cg)-Clec9atm2.1(icre)Crs/J (45);), CD11c-Cre (B6.Cg-Tg(Itgax-cre)1-1Reiz/J (46);), or Lck-Cre (Tg(Lck-cre)1Jtak (47)) mice. Mincle^tg^ mice (48) were obtained from Sho Yamasaki and crossed with Mincle KO mice (Clec4e^tm1.1Cfg^) generated and provided by the Consortium for Functional Glycomics (49). *Tnf^-/-^* mice (50) were provided by Dr. Ulrike Schleicher. C57BL/6N mice were purchased from Charles River Laboratories. At the end of the experiment the mice were euthanized by cervical dislocation. All mouse experiments were approved by the *Regierung von Unterfranken* (protocol number 55.2.2-2532-2-1641).

IL-17A fate reporter mice were made by crossing the Il17a^tm1.1(icre)Stck^/J (IL-17cre) strain with the B6.129X1-Gt(ROSA)26Sor^tm1(EYFP)Cos^/J (R26R-EYFP) strain (51) (from The Jackson Laboratory (Bar Harbor, USA)) and handled at the experimental animal facility at Statens Serum Institut. At the end of the experiment the mice were euthanized by cervical dislocation. Experimental work was conducted in accordance with the regulations of the Danish Ministry of Justice and the Danish National Experiment Inspectorate under permit 2017-15-0201–01363 and in compliance with the European Community Directive 2010/63 EU for the care and use of laboratory animals.

### Immunizations

To study the cellular immune response, we used a 7-day immunization protocol, as previously described (26, 34, 52). Mice were immunized s.c. in both footpads with 50 µl CAF01 (23) mixed with 1 µg H1 (43) per foot, except in the IL-17A fate-reporter mice, where s.c. immunization was performed at the base of tail using a 100 μl volume. Unimmunized mice were injected with the same volume of PBS. All footpad immunizations were performed on unconscious animals after inhalation anesthesia with 4% Isoflurane. For the immunization in the base of tail (s.c.) no anesthesia was used. After footpad immunization, the footpad thickness was measured before the immunization (day 0) and every second day after immunization. The footpad swelling was calculated by subtracting the initial thickness (on day 0) from the later obtained values. On day 7 after immunization, the mice were killed, and the inguinal and popliteal lymph nodes as well as the spleen were isolated.

For the experiments requiring TNF blockade, Etanercept (3 mg/kg in 100 µl PBS) was injected s.c. in the flank (without anesthetization) on the day of immunization and every other day thereafter. The control group was injected in parallel with 100 µl PBS.

For the depletion of NK1.1-positive cells, an anti-NK1.1 antibody (clone PK136, Leinco Technologies) was used in addition to etanercept treatment in the 7-day immunization protocol. 250 µg/mouse of this antibody or an isotype control (clone C1.18.4, Leinco Technologies) was injected i.p. (without anesthetization) one day before and a second time two days after immunization with H1/CAF01.

To study the humoral immune response, a 5-week immunization protocol was used. Here, the mice received a second immunization at day 21 (2 µg H1 in 100 µl CAF01, s.c. base of tail). Blood was collected at day 0 (no H1-specific Ab detectable, data not displayed) and after the killing on day 36.

To assess the role of TNF on the Ab production, additionally etanercept (3 mg/kg in 100 µl PBS) was injected s.c. in the flank on the day of immunization and every other day until day 18. Then, to prevent hindrance of the TNF block by the formation of anti-etanercept Ab, we switched to injecting anti-murine TNF Ab (200 µg/mouse, clone TN3-19.12, Leinco Technologies) every second day starting from day 20. A control group was injected with a matching isotype control (clone PIP, Leinco Technologies). All s.c. and i.p. injections of antibodies and etanercept were performed without anesthetization. In this protocol, the mice were killed 1 week after the booster immunization and blood was collected.

### Differentiation and stimulation of BMDM

Bone marrow cells were isolated from C57BL/6 mice and Mincle^-/-^; Mincle^tg^ mice and differentiated as described before (34). TDM was dissolved in isopropanol and coated to the bottom of the well (final concentration of 2 µg/ml) by allowing the isopropanol to evaporate. For the control wells, the same volume of isopropanol was evaporated. The BMDM were seeded in a flat-bottom 96-well plate (2*10^5^ cells/well). Etanercept 100 µg/ml was added to the respective wells. The cells were stimulated for 24 h.

### Isolation and stimulation of BM monocytes

To isolate monocytes from the bone marrow, the monocyte Isolation Kit (Miltenyi Biotec, Order No.: 130-100-629) was used according to the instructions of the producer. BM monocytes were stimulated by seeding them into a flat-bottom 96-well plate (2*10^5^ cells/well). The wells were coded before with isopropanol, TDB (5 µg/ml), or TDM (2 µg/ml) as described above. Etanercept (final concentration 100 µg/ml) was added to the respective wells. The cells were stimulated for 24 h.

### Restimulation of cells from spleen and lymph node

A single cell suspension of lymph node cells was obtained by mechanically dissociating the LN using a 70 µm cell strainer. The spleen was also mashed through a 70 µm strainer, and additionally, the erythrocytes were lysed using ammonium chloride. 5 x 10^5^ LN and spleen cells were restimulated in a 96-well U-bottom cell culture plate for 96h with H1 (1 µg/ml).

### Cytokine ELISA

Cytokines were measured in the supernatants of restimulated cells. For this purpose, the following ELISA kits were used according to the manufacturer’s instructions: IFNγ (R&D Systems, DY485), IL-17 (R&D Systems, DY421), IL-10 (R&D Systems, DY417), and IL-6 (R&D Systems, DY406).

### Flow cytometry

After blocking with anti-mouse CD16/32 antibody (eBioscience) the dead cells were stained with the Fixable Viability Dye eFluor 506 (Invitrogen 65-0866-14). Next, fluorophore-coupled antibodies were used to stain for the surface markers (see **Supplementary Table S1** for a list of the used antibodies). For the staining of Mincle, an uncoupled primary antibody (MBL, clone 4A9) was used and detected by using a suitable fluorescent-labeled secondary antibody.

For intracellular cytokine staining, 1.5 x 10^6^ cells were restimulated in a 96-well plate using 5 µg/ml of each of the three H1 peptides Ag85B _p241-255_, Ag85B _p261-280_, ESAT-6 _p1-15_ (obtained from Peptides and Elephants) or using the whole H1 protein. The cells were stimulated for one hour, followed by five hours in the presence of Brefeldin (10 µg/ml). After surface staining and fixation with 1% PFA overnight at 4 °C, the cells were permeabilized by saponin, and antibodies for the intracellular staining were added.

Flow cytometry measurements were performed using an LSRFortessa (BD Bioscience, 5-laser configuration, Model No. 647794E6). The flow cytometry data were analyzed using FlowJo (BD Live Sciences, v. 10.7.1).

We defined the cell types by the following marker (gating strategy in **Supplementary Figure S3**): B cells (CD19^+^, CD3-), neutrophils (CD3^-^, CD19^-^, NK1.1^-^, Ly6C^+^, Ly6G^+^), classical monocytes (CD3^-^, CD9^-^, NK1.1^-^, Ly6G^-^, CD11b^+^, Ly6C^high^), Ly6C^low^ monocytes (CD3^-^, CD19^-^, NK1.1^-^, Ly6G^-^, CD11b^+^, Ly6C^low^), NK cells (CD3^-^, CD19^-^, NK1.1^+^), NK T cells (CD3^+^, CD8^-^, CD4^-^, NK1.1^+^), macrophages (CD3^-^, CD9^-^, NK1.1^-^, Ly6G^-^, CD11b^+^, Ly6C^low^, F4/80^+^), cDC1 (CD3^-^, CD19^-^, NK1.1^-^, CD88^-^, CD26^+^, CD64^-^, MHCII^+^, CD11c^+^, XCR1^+^), cDC2 (CD3^-^, CD19^-^, NK1.1^-^, CD88^-^, CD26^+^, CD64^-^, MHCII^+^, CD11c^+^, XCR1^-^) CD4 T cells (CD3^+^, CD19^-^, γδ TCR^-^, CD4^+^, CD8^-^), CD8 T cells (CD3^+^, CD19^-^, γδ TCR^-^, CD8^+^, CD4^-^), γδ T cells (CD3^+^, CD19^-^, γδ TCR^+^) Th17 cell (CD3^+^, CD4^+^, CD8^-^, IFNγ^-^, IL-17^+^), Th1 cell (CD3^+^, CD4^+^, CD8^-^, IFNγ^+^, IL-17^-^).

### RNA isolation and qPCR

Cells were lysed using Tri reagent (Bio&Sell, order No: BS67.211.0100). The RNA was isolated using the Direct-zol™ -96 RNA isolation Kit (ZymoResearch, cat. No.: R2056) according to the instructions of the producer. The RNA was transcribed to cDNA using the High-Capacity cDNA Reverse Transcription Kit (Applied Biosystems, cat No.: 4368814).

To perform the qPCR, primers and probes from the KiCqStart^®^ Probe Assay (Sigma-Aldrich) were used for *Clec4e (Mincle)* and *Hprt* (sequence see **Supplementary Table S2**). For *Il6* and *Il1b*, the primers were used together with a probe from the Roche UPL universal probe library (sequence see **Supplementary Table S2**). Gene expression was normalized using the *Hprt* expression, and the fold change was calculated using the 2^-ΔΔct^ method (53), with isopropanol-treated samples serving as calibrators.

### Serum collection and H1-specific Ab ELISA

Blood was collected at the end of the experiment by retroorbital bleeding from unconscious animals after i.p. application of Ketamin (100 µg/g bodyweight) together with Xylazin (10 µg/g bodyweight). Afterwards, still under narcosis, the mice were killed by cervical dislocation. Sera were separated from the blood by centrifugation (900 g, 10 min). H1 (400 ng/ml) was adsorbed overnight onto an ELISA plate (Sarstedt, ref: 82.1581.200). The non-bound H1 was discarded, and after BSA block (1% BSA in PBS), the sera were applied at different dilutions. The sera were washed away and the bound H1-specific Abs were detected using the following HRP-coupled secondary Ab: goat anti-mouse IgG1 (Southern Biotech, 1071-05, 1:4000), goat anti-mouse IgG2c (Southern Biotech, 1078-05, 1:4000), goat anti-mouse IgG2b (Southern Biotech, 1091-05, 1:4000).

### Statistics

For statistical analysis, GraphPad Prism (GraphPad Software, LLC, version 9.5.1) was used. The used statistical test for every graph is indicated in the figure legends. In all graphs, each dot corresponds to one mouse, the height of the bar indicates the mean. For the two-way ANOVA tests the normal distribution of the data was tested using the Shapiro-Wilk test. If the requirement of the normal distribution was not meet, the ANOVA was performed on log transformed values, this is indicated in the figure legend.

The analysis of Ab titers was only done for immunized groups. To this end, a correlation was modeled between the measured OD values and the respective dilution step. For this, a three-parameter non-linear regression with the equation *OD = Bottom + (Top-Bottom)/(1+(titer/mid-titer point))* was used. The mid-titer point was obtained together with its SEM, and to compare these values between different groups, a one-way ANOVA followed by Dunnett’s multiple comparison test was performed (or Mann-Whitney U test in case of two groups).

The p-values of all tests are displayed on the respective comparisons; to maintain clarity, only meaningful comparisons are shown in the graphs.

## Results

### TNF blockade selectively prevents the Th17 but not the Th1 response to immunization

**Figure 1:**
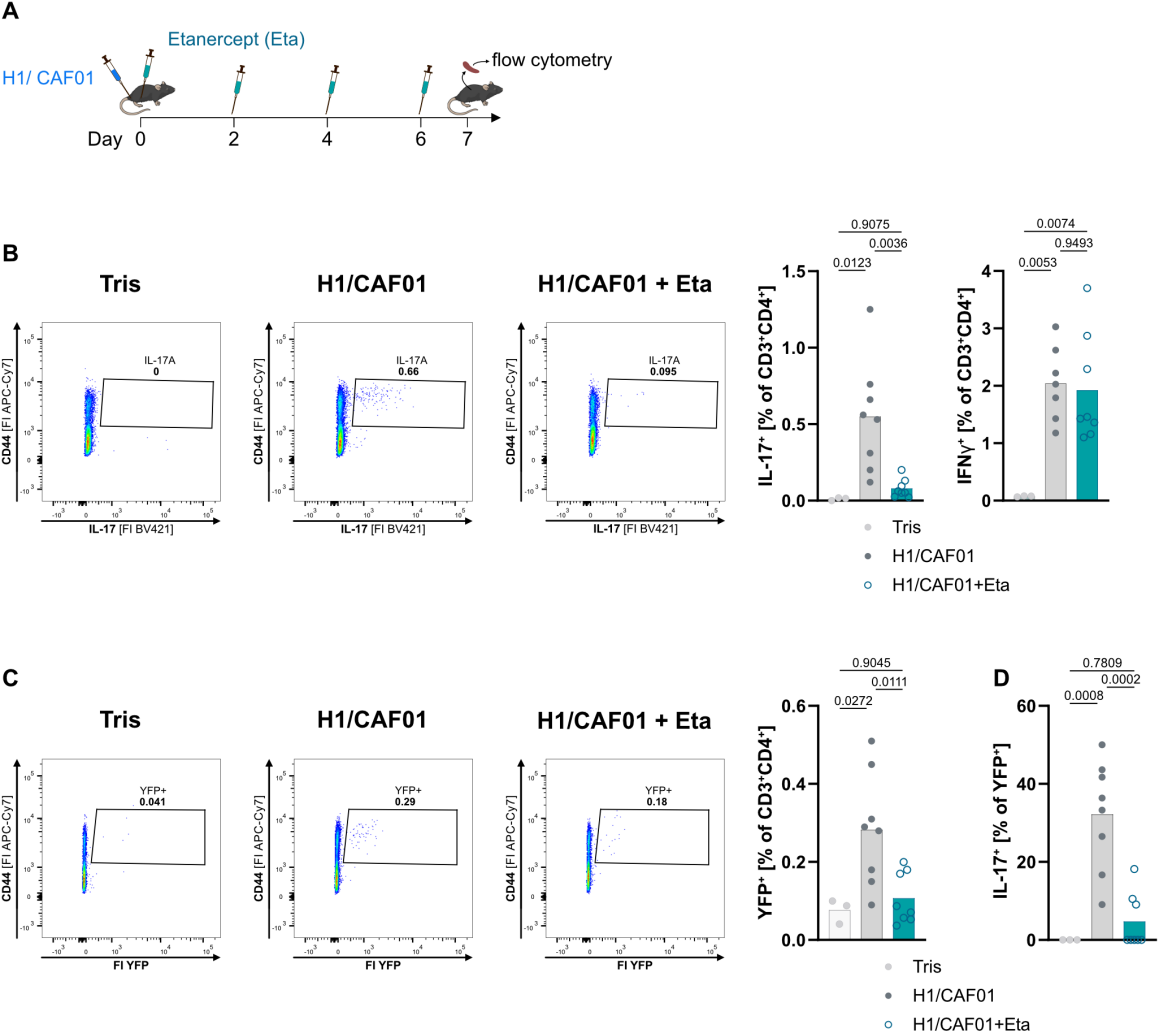
Etanercept selectively prevents the induction of H1-specific Th17 cells. IL-17A fate reporter mice, in which cells that have transcribed *Il17a* at any time will continuously express Yellow Fluorescent Protein (YFP), left unimmunized (Tris) or were immunized s.c. at the base of the tail with the antigen H1 together with the adjuvant CAF01. The mice were killed 7 days after the immunization. In one group, TNF was blocked by injecting Etanercept (Eta) every second day, whilst the control group received PBS injections **(A)**. H1-specific Th17 cells and Th1 cells in the spleen were measured via flow cytometry staining for intracellular IL-17 or IFNγ production, respectively, after H1 restimulation **(B)**. The induction of Th17 cells measured by YFP expression **(C)**. YFP+ cells were tested for the ability to produce specific IL-17 **(D)**. Depicted is one representative experiment out of two (n = 3–8 mice per group), one-way ANOVA followed by Tukey’s multiple comparison test.

We previously demonstrated that TNF is required for antigen-specific IL-17 production in mice vaccinated with the TB antigen H1, combined with the Mincle-dependent adjuvant CAF01 (34). Here, we immunized mice using the same antigen/adjuvant system while blocking TNF with Etanercept (see **Figure 1A**), restimulated splenocytes with H1 antigen and analyzed production of IL-17 and IFNγ by CD4^+^ T cells using intracellular cytokine staining (**Figure 1B**). While less than 0.1% of all CD4+ T cells from naïve mice produced IFNg or IL-17 upon stimulation with H1, the frequency of H1-specific IFNg+ Th1 cells and IL-17+ Th17 cells was around 2% and 0.6%, respectively, seven days after a single s.c. injection of CAF01/H1 (**Figure 1B**; gating strategy shown in **Supplementary Figure S1A**). The frequency of IL-17-producing cells among activated CD44+ CD4+ cells is approximately 3% (**Supplementary Figure S1B**), which is similar to previous results in a comparable antigen/adjuvant system after repeated immunization (54). Consistent with our previous ELISA-based results, the frequency of antigen-specific Th17 cells was reduced by TNF blockade during immunization, whereas the H1-specific Th1 response remained unaffected (**Figure 1B**; gating strategy shown in **Supplementary Figure S1**).

We next sought to understand whether TNF is necessary to induce or to maintain antigen-specific Th17 cells. To address this question, we utilized Th17 fate reporter mice, in which IL-17A expression results in Cre recombinase-mediated constitutive YFP expression (51). Indeed, while only few YFP^+^ cells were detected in spleens of naive mice (**Figure 1c**), CAF01/H1 induced a significant increase of YFP^+^ cells, to a percentage comparable to the frequency of IL-17A^+^ cells (0.3 vs. 0.5%). In mice immunized under TNF blockade, the number of YFP-marked cells decreased to the level found in unimmunized mice (**Figure 1c**). In mice immunized without Etanercept, around 30% of YFP-marked cells produced IL-17 after H1 re-stimulation. The YFP-marked cells detected in mice treated with TNF blockade were unable to produce IL-17 after specific restimulation, comparable to unimmunized mice (**Figure 1D**). This small percentage of YFP+ cells that are non-reactive to H1 likely represents Th17 cells with other antigen specificities. Thus, we conclude that TNF is essential for inducing the differentiation of naïve T cells into Th17 cells in the H1/CAF01 vaccination model.

### Preventing TNF signaling via TNFR1 on myeloid cells reduces the local inflammatory response towards the adjuvant

TNF affects multiple target cell types and exerts diverse biological effects. It signals through TNFR1 and TNFR2, with TNFR1 being more crucial for its pro-inflammatory function (1). We aimed to determine which immune cell type requires TNFR1 signaling for the induction of Th17 cells. Considering TNFR1’s vital role in upregulating the PRR Mincle on macrophages (34), we hypothesized that TNF facilitates the recognition of the adjuvant by antigen-presenting and other innate immune cells. Alternatively, or in addition, an effect on B or T cells could explain the requirement for TNF in the H1/CAF01 vaccination model.

**Figure 2:**
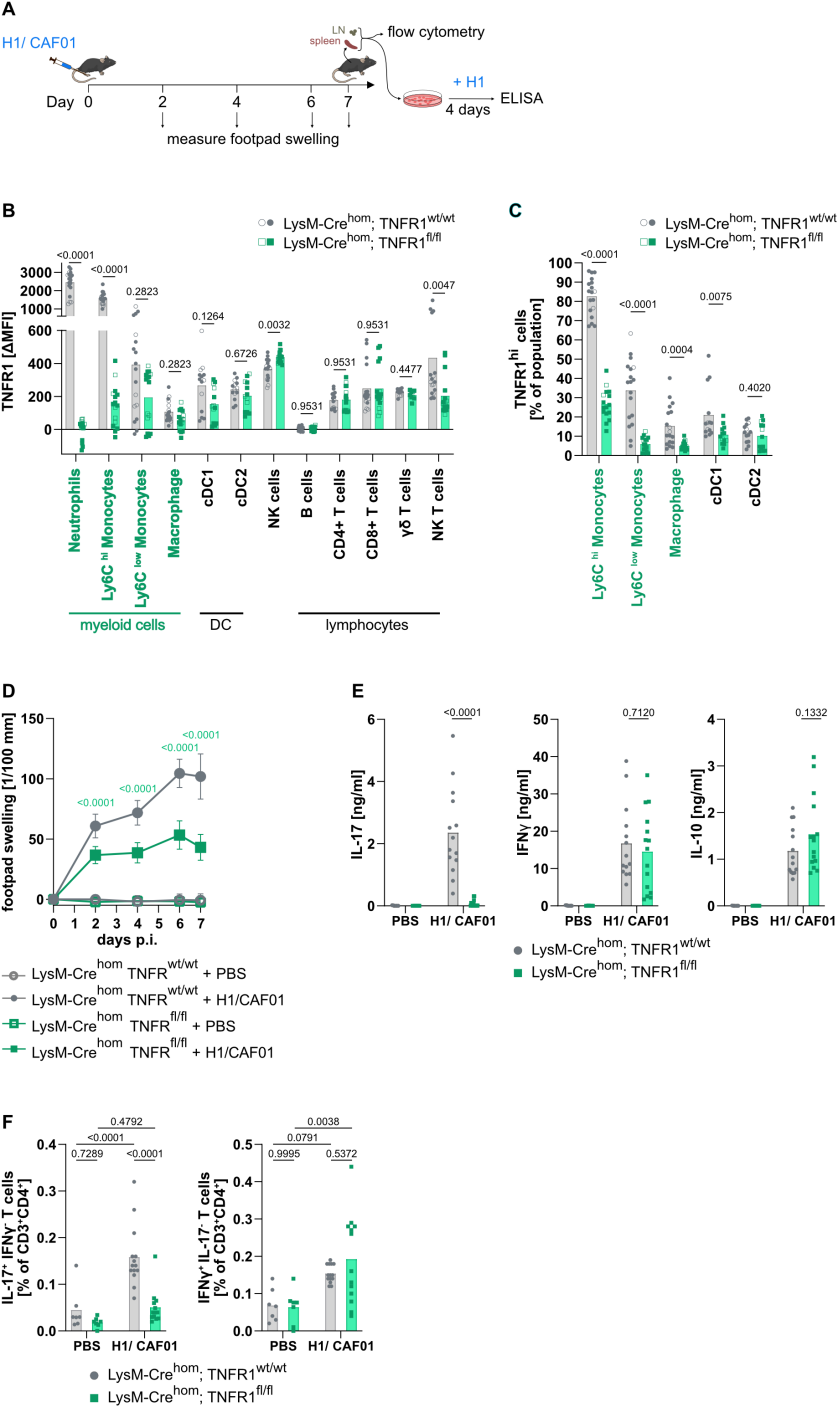
Deletion of TNFR1 on myeloid cells prevents induction of specific Th17 cells after H1/CAF01 immunization. LysM-Cre^hom^; TNFR1^fl/fl^ and LysM-Cre^hom^; TNFR1^wt/wt^ mice were immunized s.c. in the footpad with H1 combined with the adjuvant CAF01 (H1/CAF01) or injected with PBS **(A)**. The mice were killed 7 days after immunization. Cell surface TNFR1 on spleen cells was measured by flow cytometry as difference in the median fluorescent intensity (ΔMFI), comparing the signals obtained using anti-TNFR1 and isotype control antibodies. Data from immunized (filled symbols) and unimmunized mice (open symbols) of each genotype were combined **(B)**. For some cell types, a bimodal expression of the TNFR1 was observed. For these cell types, the percentage of highly TNFR1-positive cells was additionally compared. Data from immunized (filled symbols) and unimmunized mice (open symbols) of each genotype were combined **(C)**. Footpad swelling was measured during the experiment **(D)**. Cells from the injection site-draining LN were isolated, and secreted cytokines were measured by ELISA four days after restimulation with H1 **(E)**. The amount of IL-17 or IFNγ-producing Th cells in the draining LN was measured after H1 peptide restimulation using flow cytometry **(F)**. Pooled from three independent experiments (n = 7–15 mice per group in total), Mann-Whitney U tests with Holm-Sidak multiple comparison correction **(B, C)**, three-way ANOVA, followed by Tukey’s multiple comparison test, displayed is the Mean with SD **(D)**, or two-way ANOVA followed by Sidak’s multiple comparison test **(E, F)**. Only the significance levels between the immunized groups at one time point **(D)** or between immunized groups **(E)** were displayed.

We utilized cell type-specific deletion of TNFR1 to dissect the mechanistic cellular targets. We initially measured TNFR1 surface expression on immune cells from wild type (WT) mice 7 days after immunization (experimental scheme in **Figure 2A**) and without immunization (**Figure 2B**; **Supplementary Figure S3**). We observed no significant TNFR1 expression on B cells. Consequently, we did not further pursue the role of TNFR1 signaling in B cells. TNFR1 was most highly expressed on neutrophils and classical monocytes, while Ly6C^low^ monocytes expressed the receptor at a lower level and only on a subset (**Supplementary Figure S3A**). NK cells and NK T cells expressed TNFR1 at a level comparable to Ly6C^low^ monocytes. cDC1 (CD88^-^, CD26^+^, CD64^-^, MHCII^+^, CD11c^+^, XCR1^+^) and cDC2 (CD88^-^, CD26^+^, CD64^-^, MHCII^+^, CD11c^+^, XCR1^-^) as well as T cells (CD4 T cells, CD8 T cells and γδ T cells) showed a lower expression level. On macrophages (Ly6G^-^, CD11b^+^, Ly6C^low^, F4/80^+^) only a small subpopulation showed a detectable TNFR1 expression.

First, we checked the importance of TNF signaling via TNFR1 on myeloid cells. To this end, we crossed LysM-Cre mice (44) with mice in which exons 2–5 of the TNFR1 are floxed (Tnfrsf1a^tm3.3Gkl^ (41),). We immunized the LysM-Cre^hom^; TNFR1^fl/fl^ mice with H1/CAF01 and determined the TNFR1 expression 7 days after immunization via flow cytometry (**Figure 2A**). LysM-Cre-mediated deletion of the TNFR1 resulted in the complete loss of TNFR1 expression on neutrophils and a substantial reduction on Ly6C^high^ inflammatory monocytes (**Figure 2B**). On Ly6C^low^ monocytes and on macrophages, TNFR1 median fluorescence intensity (MFI) was not reduced significantly by LysM-Cre mediated deletion. Closer examination of the flow cytometry data showed that TNFR1 was expressed on subpopulations of Ly6C^low^ monocytes and F4/80^+^ macrophages (**Supplementary Figure S3A**). LysM-Cre strongly reduced TNFR1 staining in these subpopulations, decreasing TNFR1^high^ cells from 34% to 6% for Ly6C^low^ monocytes and from 15% to 5% for macrophages (**Figure 2C**). For cDC1 the percentage of TNFR1^high^ cells was significantly reduced by approximately 50% (**Figure 2C**). In all other tested cell types, the MFI of the TNFR1 expression was not significantly changed, except for a slight increase on NK cells and a reduction on NK T cells. We will address the potential importance of TNFR1 on cDC1, NK and NK T cells later.

We first studied the effect of TNFR1 deficiency in myeloid cells on the local inflammatory response to immunization with H1/CAF01 (**Figure 2A**). CAF01, a liposome-based adjuvant, forms a depot at the site of injection (55, 56). Innate immune cells migrate to this location and slowly transport the adjuvant and antigen to the draining lymph node (57). Upon s.c. immunization in the footpad, the measurement of its thickness over time can serve as a proxy for immune cell infiltration and inflammation at the site of injection. The robust footpad swelling after H1/CAF01 injection was reduced by approximately 50% in myeloid TNFR1 knockout (KO) mice compared to the control group (**Figure 2D**), which corresponds well to the reduction observed in *Tnf*^-/-^ mice (34), indicating that TNF signaling in myeloid cells plays a significant role in the local inflammatory response.

### TNFR1 signaling in myeloid cells is essential for the antigen-specific Th17 response

The absence of TNFR1 in myeloid cells completely prevented the induction of H1-specific production of IL-17 by LN cells, while the production of IFNγ and IL-10 remained unaltered (**Figure 2E**; similar results observed for spleen cells see **Supplementary Figure S4A**). Of note, neither the floxing of TNFR1 alone (**Supplementary Figure S5A**) nor the insertion of the Cre recombinase under the LysM promoter (**Supplementary Figure S5B**) influenced the IL-17 production after immunization. The reduced IL-17 production was accompanied by the vaccine’s inability to induce H1-specific Th17 cells in the myeloid TNFR1 KO mice, whereas the induction of Th1 cells was unaffected (data from LN in **Figure 2F**, comparable results were observed for spleen **Supplementary Figures S4B, C**). In summary, the absence of TNFR1 on myeloid cells abrogated the Th17 response induced by the Mincle-activating adjuvant CAF01, replicating the effect of genetic whole body deletion of the *Tnf* gene we showed before (34).

### TNF contributes to antibody induction, but TNFR1 on myeloid cells is dispensable

We also tested the consequence of genetic deletion or pharmacologic blockade of TNF versus the specific knockout of TNFR1 in myeloid cells for the induction of the humoral immune response. To do so, we measured H1-specific antibody (Ab) titers after two injections of H1/CAF01 (Experimental layout **Supplementary Figure S2A**). Knockout of TNF reduced titers of all studied isotypes by more than one order of magnitude (**Supplementary Figure S2B**). This reduction was not surprising, as it is well established that the formation of germinal centers as well as the production of isotype switched antibodies, is impaired in *Tnf^-/-^* and *TNFR1^-/-^* mice (50, 58). Transient pharmacological TNF blockade of the response to vaccination by sequential treatment with Etanercept and a neutralizing anti-TNF antibody caused a weaker but significant reduction in H1-specific Ab titers of the proinflammatory isotypes IgG2c and IgG2b, but not of IgG1 (**Supplementary Figure S2B**). In contrast to the effect of genetic deletion or pharmacological inhibition of TNF, the myeloid cell-specific TNFR1 KO did not affect the induction of H1-specific Ab after immunization (**Supplementary Figure S2C**).

### TNFR1 on DC plays a limited role in the antigen-specific Th17 response and is dispensable for induction of H1-specific antibodies

**Figure 3:**
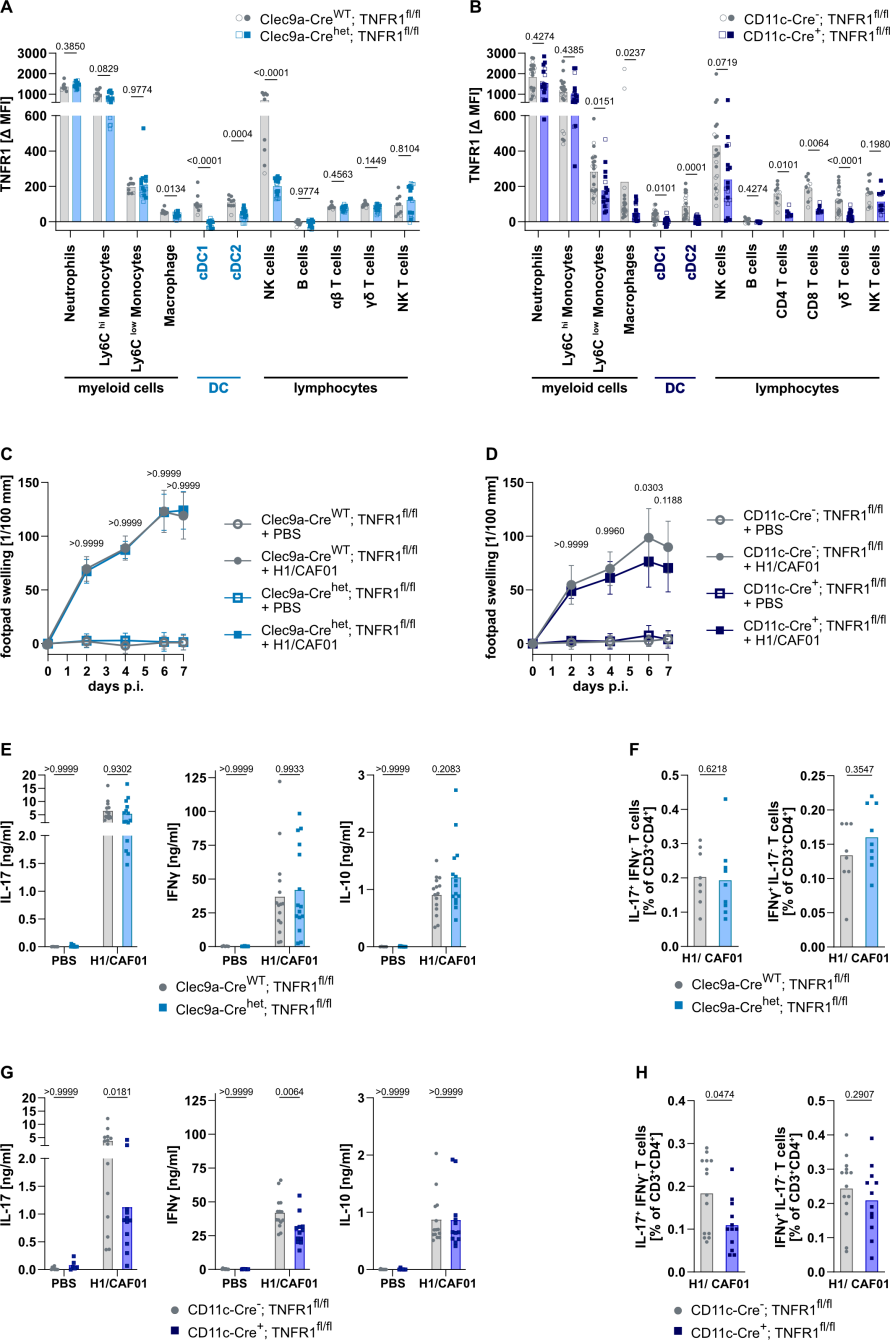
TNFR1 on DC plays a limited role in the Th17 response after H1/CAF01 immunization. Clec9a-Cre^het^; TNFR1^fl/fl^ and CD11c-Cre^+^; TNFR1^fl/fl^ mice as two different DC deletion models, and TNFR1^fl/fl^ mice as controls, were used. Mice were immunized s.c. in the footpad with H1/CAF01 and killed 7 days later. Expression of TNFR1 was measured by flow cytometry on spleen cells to detect deletion efficiency mediated by Clec9a-Cre **(A)** and CD11c-Cre **(B)** mediated deletion of the TNFR1. Data from immunized (filled symbols) and unimmunized mice (open symbols) of each genotype were combined. Footpad swelling was measured **(C, D)**. Cells from the draining LN were isolated, and secreted cytokines were measured by ELISA 4 days after restimulation with H1 protein for the Clec9a-Cre **(E)** and CD11c-Cre **(G)** deletion models. The amount of IL-17 or IFNγ-producing Th cells from LN was measured after H1 peptide restimulation using flow cytometry for Clec9a-Cre **(F)** and CD11c-Cre **(H)** mediated deletion. For the data shown in F and H, the restimulation of unimmunized cells was impossible due to a lack of cells. From spleen cells, similar results were obtained, and here we were able to include this condition (**Supplementary Figure S8B** and D). Pooled from three independent experiments (n = 6–14 mice per group in total). Mann-Whitney U tests with Holm-Sidak multiple comparison correction **(A, B)**, three-way ANOVA, followed by Tukey’s multiple comparison test, with the mean and SD displayed **(C, D)** or two-way ANOVA, followed by Sidak’s multiple comparison test **(E, G)** or Mann Whitney U test **(F, H)**. Only the significance levels between the immunized groups at one time point **(C, D)** or within immunization groups **(E, G)** are displayed.

After we found that TNFR1 on myeloid cells is essential for the Th17 response, we wanted to test the importance of the TNFR1 on DCs. For this, we employed two well-characterized DC-targeting Cre-deleter mouse lines. First, a Cre knock-in at the Clec9a locus specifically deletes *loxP*-flanked genes in DC progenitors (45). In Clec9a-Cre^het^; TNFR1^fl/fl^ mice, we observed a complete absence of TNFR1 staining on cDC1 and a significant, but incomplete reduction (ca. 65%) of TNFR1 surface protein on cDC2 (**Figure 3A**, representative histograms **Supplementary Figure S6A**). Besides DCs, there was a reduction of TNFR1 expression on macrophages and on NK cells, whilst the remaining tested cell types had intact TNFR1 expression. We will address the potential role of NK cells in the induction of antigen-specific Th17 cells later in this manuscript.

To achieve efficient deletion of TNFR1 in both cDC subsets, we used CD11c-Cre-mediated deletion (46) of TNFR1 in addition. In this model, TNFR1 staining was completely absent on cDC1 and cDC2 (**Figure 3B**; representative histograms **Supplementary Figure S6B**). In contrast to the Clec9a-Cre model, there was also a reduction in TNFR1 surface protein across all T cell subsets. Additionally, a significant reduction of TNFR1 was observed in Ly6C^low^ monocytes and in macrophages.

In both models for deleting TNFR1 on DC, the inflammatory response at the site of injection, measured by the footpad swelling, was unaffected (**Figures 3C, D**). Clec9a-mediated TNFR1 deletion did not alter cytokine production after the restimulation of LN cells from immunized mice (**Figure 3E**). These findings were confirmed by the unaltered induction of Ag-specific Th17 and Th1 cells after immunization (**Figure 3F**). This suggests that TNFR1 expression on cDC1 cells is not required for H1/CAF01-induced Th17 responses. Therefore, the complete loss of IL-17 production and Th17 cell induction LysM-Cre-mediated deletion (**Figures 2E, F**) cannot be caused by the reduction of TNFR1^high^ cDC1 observed in **Figure 2C**.

The CD11c-Cre-mediated complete TNFR1 deletion on all DC subsets did not affect the production of IFNγ and IL-10 in LN cells (**Figure 3G**) nor the induction of Th1 cells (**Figure 3H**). In contrast, it led to a significant reduction in IL-17 production upon restimulation of LN cells with H1, with 75-80% reduction when measured by ELISA (**Figure 3G**), or around 50% reduction in LN CD4+ T cells producing IL-17A, as measured by intracellular cytokine staining (**Figure 3H**). The same pattern was observed after restimulation of spleen cells and measurement by ELISA (**Supplementary Figures S8A, C**) and after intracellular cytokine staining of spleen cells (**Supplementary Figures S8B, D**). No significant inhibition of IL-17 production was observed in CD11c-Cre^+^; TNFR1^wt/wt^ mice (**Supplementary Figure S5C**). In summary, TNFR1 on cDC2 seems to play a role in inducing a Th17 response after H1/CAF01 immunization, but the Th17 response does not entirely depend on it, as observed for the TNFR1 on myeloid cells. Of note, CD11c-Cre-mediated deletion of TNFR1 is not entirely specific for DC but also leads to reduced expression on monocytes, macrophages and T cells.

We also measured the H1-specific Ab titer in H1/CAF01-immunized CLec9a-Cre^het^; TNFR1^fl/fl^ and CD11c-Cre^+^; TNFR1^fl/fl^ mice. In both models, the specific Ab titers were similar to those in the control group (**Supplementary Figure S2C**). This implies that TNFR1 on DC is dispensable for the H1/CAF01-induced Ab response.

### TNFR1 on T cells is not required for the antigen-specific Th17 response

The impaired Th17 response in CD11c-Cre^+^; TNFR1^fl/fl^ mice was associated with a reduction in TNFR1 surface protein on T cells (**Figure 3B**), raising the question whether TNFR1 on T cells is required for Th17 differentiation in our immunization model. To address this, we used the Lck-Cre mouse (47) to delete the floxed TNFR1 during thymic development of T cells.

**Figure 4:**
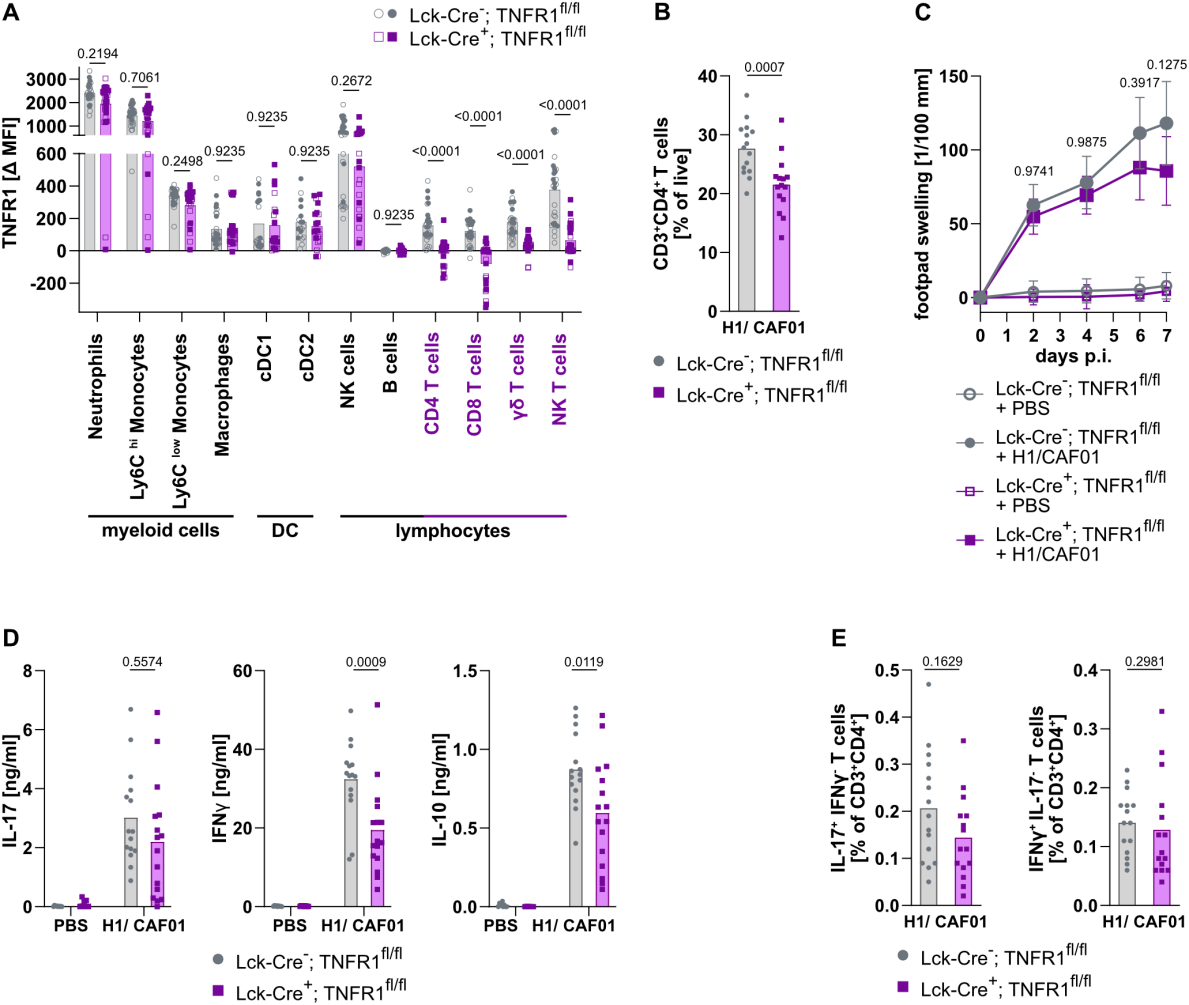
Deletion of TNFR1 in T cells does not impair Th17 cell induction. Lck-Cre^+^; TNFR1^fl/fl^ and TNFR1^fl/fl^ control mice were immunized s.c. in the footpad with H1/CAF01 and killed 7 days after immunization. The expression of the TNFR1 on spleen cells was measured by flow cytometry. Data from immunized (filled symbols) and unimmunized mice (open symbols) of each genotype were combined **(A)**. The frequency of Th cells, defined as CD3^+^ CD4^+^ cells, was measured in the LN by flow cytometry **(B)**; the restimulation of unimmunized cells was impossible due to a lack of cells. From spleen cells, similar results were obtained, and here we were able to include this condition (**Supplementary Figure S9B**). The footpad swelling was measured during the experiment **(C)**. Draining LN cells were isolated, and secreted cytokines were measured via ELISA 4 days after restimulation with H1 protein **(D)**. The amount of IL-17 or IFNγ-producing Th cells in the LN after H1 peptide restimulation was measured using flow cytometry, the restimulation of unimmunized cells was impossible due to a lack of cells **(E)**. Similar results were obtained with spleen cells (**Supplementary Figure S9C**). Pooled from three independent experiments (n = 8–16 mice per group in total), Mann-Whitney U tests with Holm-Sidak multiple comparison correction **(A)** or Mann Whitney U test **(B, E)** or three-way ANOVA, followed by Tukey’s multiple comparison test **(C)** or two-way ANOVA followed by Sidak’s multiple comparison test **(D)**. For C, the mean is displayed together with the SD. Only the significance levels between the immunized groups at one time point **(C)** or between immunized groups **(D)** were displayed.

In this model, TNFR1 was no longer present on the surface of CD4^+^ and CD8^+^ T cells as well as on γδ T cells and NK T cells (**Figure 4A**; representative example in **Supplementary Figure S7**). Besides this, TNFR1 expression on all other studied cell types was unaffected. Thus, Lck-Cre-mediated deletion was efficient and specific in the T cell compartment.

Of note, a lower frequency of CD4^+^ T cells was observed in the LN of naïve and immunized Lck-Cre^+^; TNFR1^fl/fl^ mice (**Figure 4B**; comparable results were observed for CD4+ spleen cells **Supplementary Figure S9B**). The local inflammatory response in the footpad remained unchanged (**Figure 4C**). The absence of TNFR1 did not significantly affect IL-17 production following the restimulation of LN cells from immunized mice (**Figure 4D**; comparable to spleen cells **Supplementary Figure S9A**). However, a significant reduction in IFNγ and IL-10 production was noted in the supernatants, whereas the frequency of induced specific Th17 and Th1 CD4+ cells in the LN remained unchanged (**Figure 4E**; spleen comparable see **Supplementary Figure S9B**). In conclusion, the TNFR1 on CD4+ T cells may contribute to their overall numbers in spleen, but was not essential for the induction of specific Th17 cells by the H1/CAF01 vaccine.

### The adjuvant CAF01 induced Th17-polarizing cytokines in monocytes in a TNF-dependent manner

**Figure 5:**
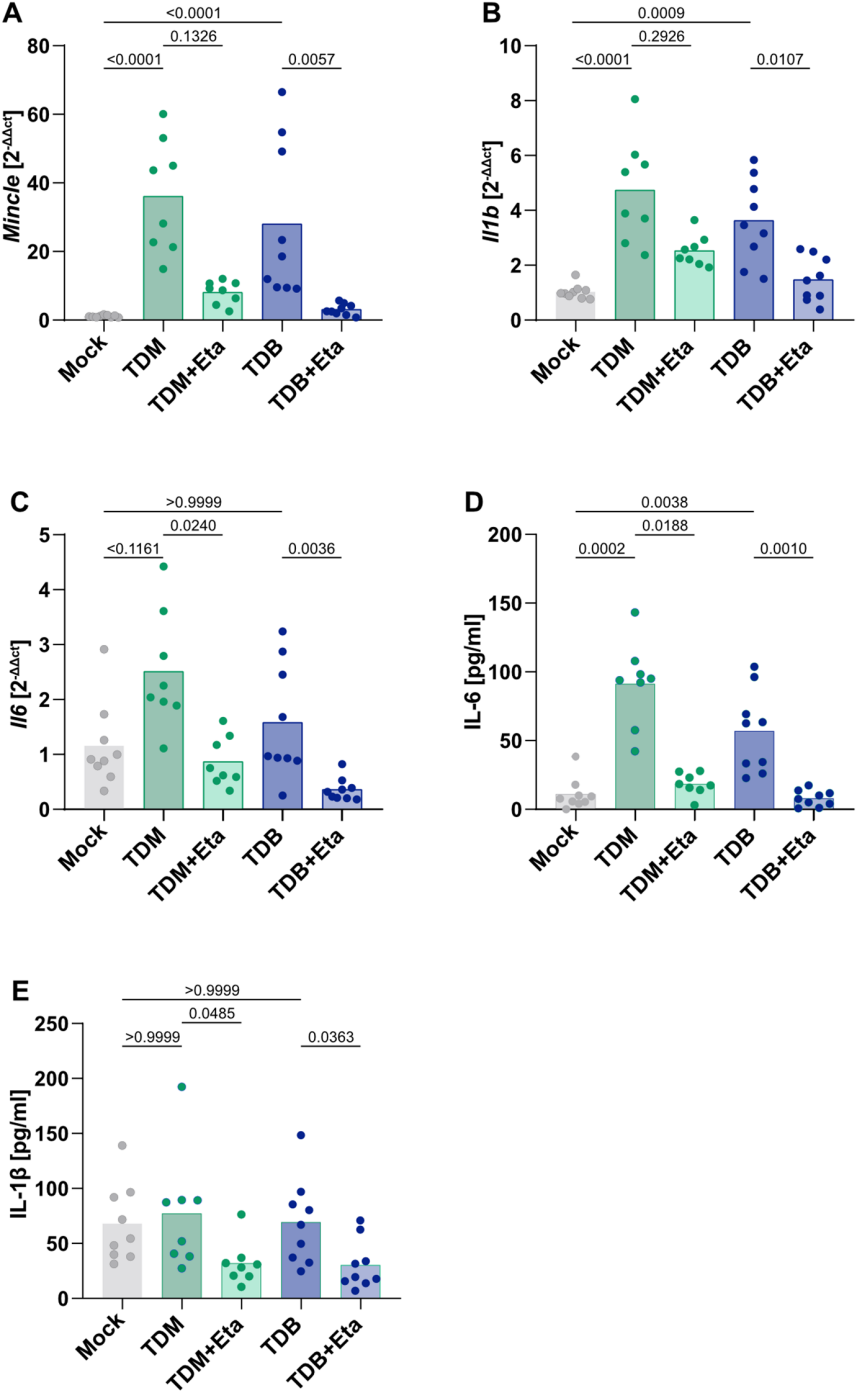
Stimulation of monocytes with TDB and TDM induces TNF-dependent upregulation of Mincle and Th17-promoting cytokines. BM monocytes were isolated and stimulated *in vitro* with TDM or TDB in the presence or absence of Etanercept (Eta). *Mincle* expression was measured after 24 hours via qPCR **(A)**; the signal obtained from unstimulated cells was used as a calibrator for the fold change calculation. *Il1b* **(B)** and *Il6* **(C)** expression was measured after 24h via qPCR, and the cytokine secretion in the supernatant was measured after 24h by ELISA for IL-6 **(D)** and IL-1β **(E)**. Pooled from three independent experiments (n = 8–9 mice per group in total), two-way ANOVA followed by Sidak’s multiple comparison test.

As TNFR1 deletion by LysM-Cre caused a complete loss of Th17 induction, we sought to dissect which myeloid cell type (monocytes, neutrophils or macrophages) needs to respond to TNF for successful vaccination. We previously found, using antibody-mediated or genetic depletion models, that CCR2^+^ monocytes are critically required for IL-17 induction by CAF01 *in vivo*, whereas an important contribution of neutrophils was excluded (59). Therefore, we now focused on the response of monocytes isolated from mouse bone marrow cells to the vaccine adjuvant and its regulation by TNF. Stimulation with the Mincle ligand TDB (contained in CAF01) as well as with the mycobacterial cord factor TDM caused a transcriptional upregulation of Mincle that was strongly reduced by the TNF blocker Etanercept (**Figure 5A**). Both Mincle ligands TDB and TDM induced the expression of *Il6* and *Il1b* in monocytes, again in a TNF-dependent manner (**Figures 5B, C**). IL-6 protein in the supernatant of monocyte cultures paralleled mRNA data (**Figure 5D**), whereas for IL-1β basal levels were unexpectedly high but still TNF-dependent after stimulation (**Figure 5E**). Thus, the CAF01 constituent TDB promotes the production of the Th17 polarizing cytokines IL-6 and Il-1β by monocytes, probably via TNF-dependent upregulation of Mincle.

### Transgenic constitutive expression of Mincle uncouples its surface expression from TNF signaling

Next, we aimed to assess whether the impaired upregulation of the TDB receptor Mincle in mice treated with Etanercept or lacking TNFR1 in myeloid cells is causal in the abrogation of Th17 induction after vaccination. To achieve this, we used a transgenic mouse model in which Mincle is constitutively expressed at a high level (Mincle^tg^ (48)). Additionally, to rule out confounding factors due to the regulation of endogenous Mincle by TNF signaling, the Mincle^tg^ mice were crossed onto a Mincle-deficient background (*Clec4e^-/^*^-^, further referred to as Mincle^-/-^ (49)).

**Figure 6:**
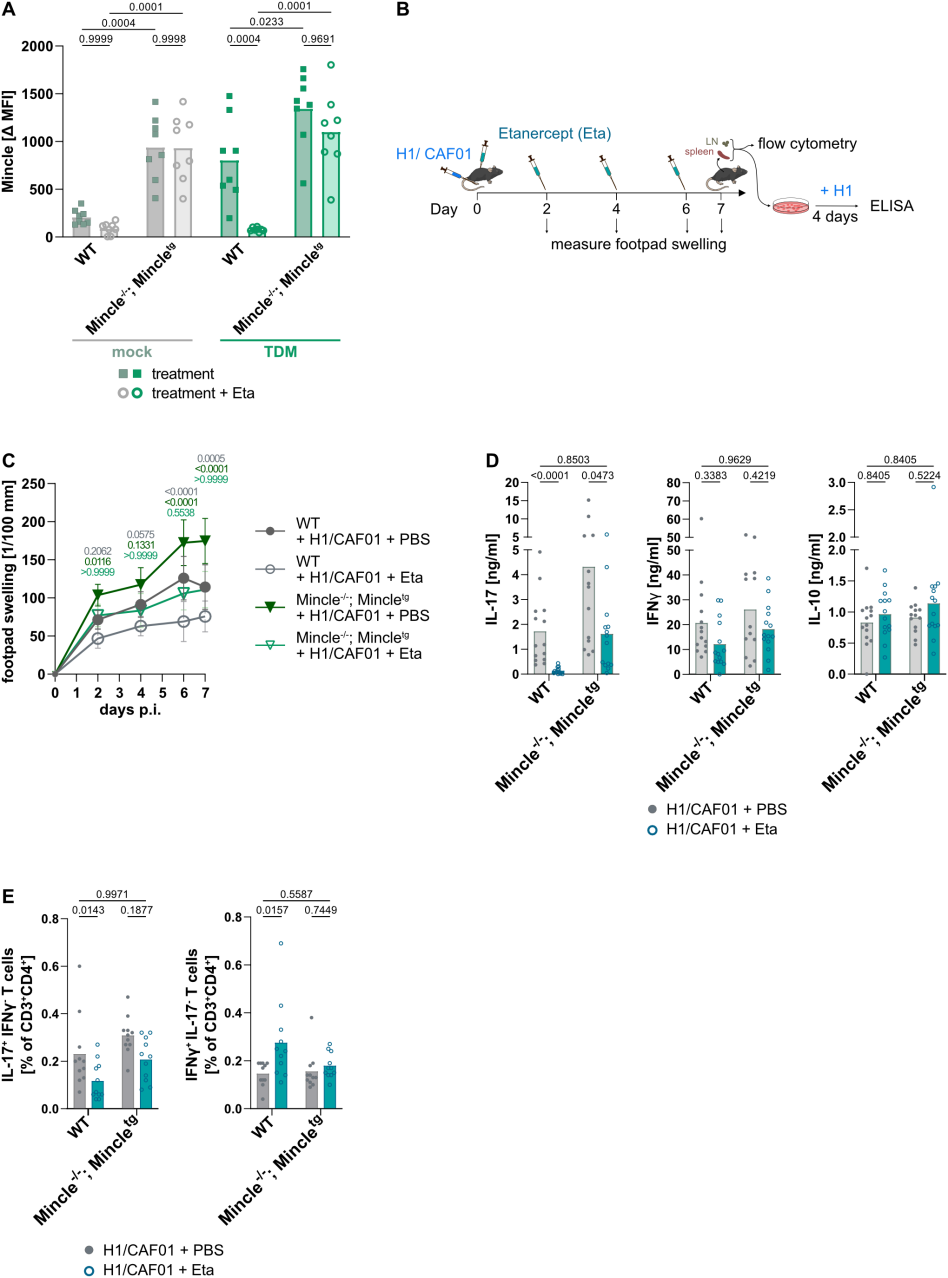
Constitutive Mincle expression antagonizes inhibition of Th17 induction by Etanercept. Mincle expression on WT and Mincle^-/-^; Mincle^tg^ BMDM was measured by flow cytometry **(A)**. The ΔMFI was calculated by subtracting the signal obtained after staining with the Mincle Ab from the signal obtained with the Isotype control. WT and Mincle^-/-^; Mincle^tg^ mice were immunized with H1/CAF01 in the presence or absence of the TNF blocker Etanercept (Eta), and mice were killed after 7 days **(B)**. The footpad swelling was measured during the experiment **(C)**. Cells from the injection site draining LN were isolated, and secreted cytokines were measured by ELISA 4 days after restimulation with H1 **(D)**. The amount of IL-17 or IFNγ-producing Th cells in the draining LN was measured after H1 peptide restimulation using flow cytometry **(E)**. Pooled from three independent Experiments (n = 13–16 mice per group in total), two-way ANOVA followed by Sidak’s multiple comparison test performed on log-transformed data **(A, D, E)**, or three-way ANOVA followed by Tukey’s multiple comparison test **(C)**. For C, the mean and SD are displayed. Only the significance levels between the immunized groups at one time point compared with the WT without Eta were displayed **(C)**.

To determine Mincle regulation in these mice, bone marrow-derived macrophages (BMDM) were generated and stimulated with the Mincle ligand TDM (**Figure 6A**; representative histograms **Supplementary Figure S10A**). Mincle was expressed at low levels on resting WT BMDM and as expected strongly upregulated in a TNF-dependent manner after stimulation. In Mincle^-/-^; Mincle^tg^ BMDM, Mincle was highly expressed under resting conditions, and, importantly, its expression level was unaffected by the TNF blocker Etanercept.

### TNF-independent, high constitutive Mincle expression can rescue Th17 induction

We immunized the Mincle^-/-^; Mincle^tg^ mice to test the effect of TNF block when Mincle expression is no longer regulated (experimental design **Figure 6B**). The local inflammatory response, measured by footpad swelling, was reduced by the TNF block in WT mice (**Figure 6C**). The magnitude of this reduction was comparable to that seen in mice lacking TNFR1 on myeloid cells (**Figure 2D**). In the immunized transgenic mice, footpad swelling was greater compared to WT mice. TNF blockade with Etanercept reduced it, but only to the level seen in immunized non-TNF-blocked WT mice (**Figure 6C**).

IL-17 production after restimulation of LN cells from immunized mice was, as expected, entirely prevented in TNF-blocked WT mice (**Figure 6D**; comparable results for spleen cells, see **Supplementary Figure S10B**). In contrast, IL-17 production was restored in TNF-blocked transgenic mice to the level observed in immunized WT mice. The production of IFNγ and IL-10 was not altered by the TNF block. Correspondingly, the frequency of specific Th17 cells was reduced in immunized TNF-blocked WT mice, whereas in Mincle-transgenic mice, the TNF blocker did not affect the frequency of Th17 cells compared to immunized WT mice (**Figure 6E**; comparable results for spleen cells, see **Supplementary Figures S10C, D**).

Under physiological conditions, Mincle is expressed on myeloid cells and some dendritic cells (DC) (28). Some reports have found it under special circumstances on B cells (29, 30) and T cells (31). We found that NK and NK T cells from Mincle^-/-^; Mincle^tg^, but not WT, mice expressed significant Mincle protein on the surface, (**Supplementary Figure S11A**). Whether NK and NK T cells contributed to the observed rescue of Th17 induction, was tested by depleting NK1.1-positive cells using the antibody PK136 (**Supplementary Figures S11B, C**). Immunization of WT mice in the absence of NK1.1-positive NK and NK T cells still led to robust IL-17 induction (**Supplementary Figure S11D**), and its complete prevention by TNF blockade. This lack of a role for NK1.1-positive cells also allows us to exclude an impact of the observed differences in TNFR1 expression on NK and NK T cells after Clec9a-Cre- or CD11c-Cre-mediated deletion (**Figures 3A, B**). Importantly, the rescue of IL-17 production in TNF-blocked Mincle^-/-^; Mincle^tg^ mice was unaffected by the absence of NK1.1-positive cells (**Supplementary Figure S11D**). Together, the Th17 rescue effect observed in the transgenic mice most likely originates from the constitutive expression of Mincle on either monocytes or DC, since NK1.1-positive cells were dispensable and transgenic Mincle was not stably expressed on T and B cells (**Supplementary Figure S11A**).

## Discussion

We employed conditional TNFR1-deficient mice to dissect the mechanism underlying our previous observation that TNF is required for Th17 induction by the Mincle-dependent adjuvant CAF01 (34). The complete loss of IL-17-producing Th cells in mice lacking TNFR1 in myeloid cells reproduced the effect of whole-body TNF deficiency. Our previous demonstration that CAF01-induced Th17 differentiation requires CCR2^+^ monocytes, but not Ly6G^+^ neutrophils (59), allows us to conclude that TNFR1 on monocytes or macrophages is most likely essential for Th17 induction. In contrast, TNFR1 deletion in DC by CD11c-Cre resulted in an incomplete reduction in IL-17-producing CD4^+^ T cells, whereas Clec9a-Cre-mediated deletion had no effect on Th cell differentiation.

**Figure 7:**
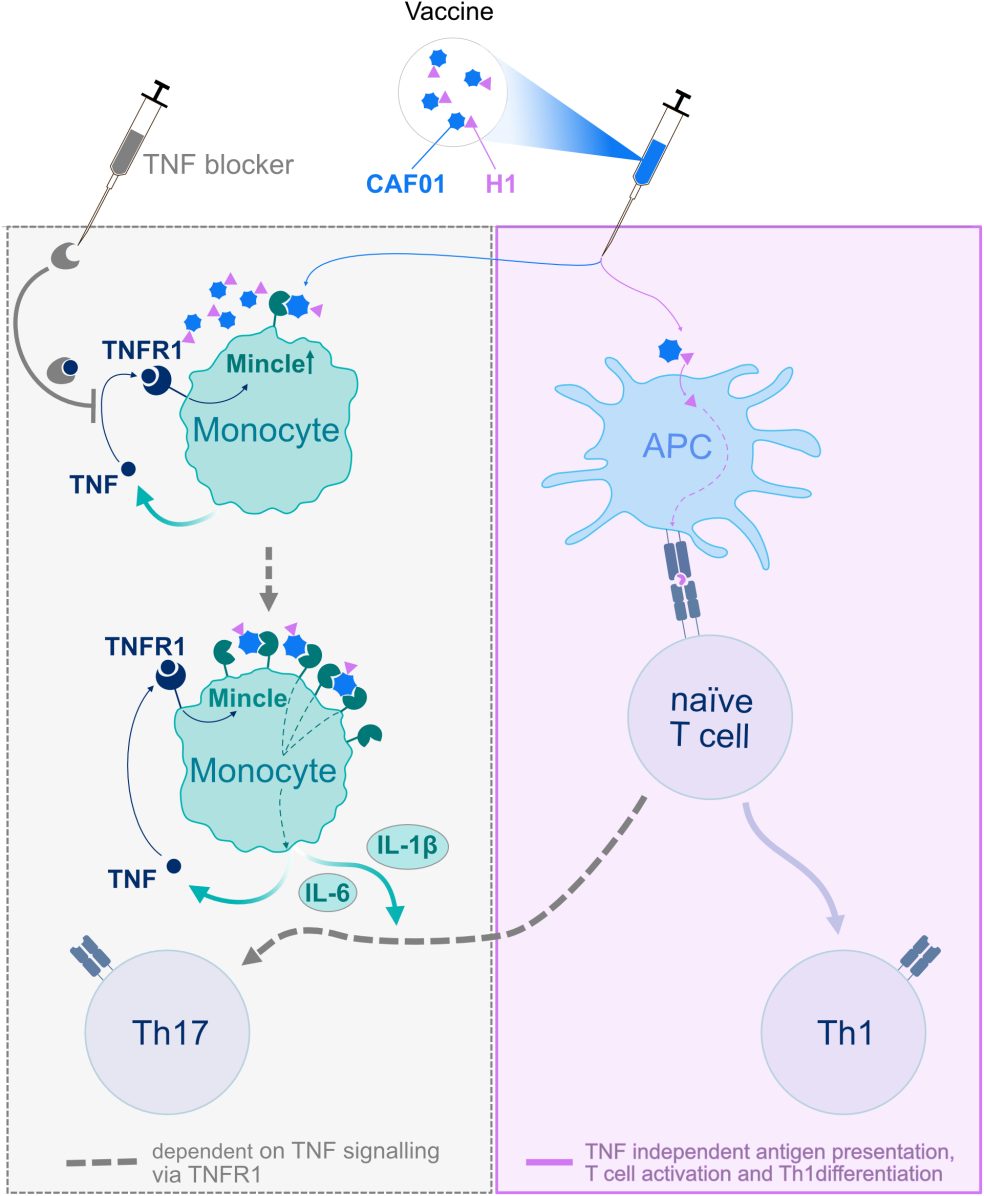
Graphical summary of the TNFR1-dependent mechanism for CAF01 adjuvanticity. The antigen H1 (purple triangle) and the adjuvant CAF01 (blue spheres) are injected together. CAF01 activates monocytes via the receptor Mincle, which is expressed at low level on resting monocytes. Activated monocytes secrete cytokines including TNF, which via TNFR1 up-regulates Mincle, resulting in enhanced sensing of CAF01 and finally the secretion of Th17 differentiating cytokines like IL1β and IL-6. These cytokines enable the differentiation of H1-specific CD4+ Th cells towards Th17 cells. TNF blockers prevent the upregulation of Mincle (dashed arrow) resulting in abrogated Th17 induction.

Finally, TNFR1 in T cells was not required for Th17 induction at all. Mechanistically, the partial restoration of Th17 adjuvanticity in Mincle^-/-^; Mincle^tg^ mice undergoing TNF blockade showed that downregulation of Mincle expression contributes causally to the loss of Th17 differentiation by impairing myeloid cell sensing of the adjuvant TDB. A graphical summary of the mechanistic role played by myeloid TNFR1 for Th17 induction is provided in **Figure 7**.

Our results indicate that TNF and TNFR1 signaling are not required for T cell priming *in vivo* by the H1/CAF01 vaccination, as the induction of antigen-specific T cells producing IFNγ or IL-10 upon restimulation was unaffected by genetic deficiency or pharmacological blockade. However, the cytokine environment in the draining lymph node and the spleen acts as signal 3, directing the differentiation of Th cells. IL-1β, IL-6, IL-23 and TGFβ promote Th17 differentiation (60), while IFNγ and IL-4 inhibit it (61). We previously showed that IL-1 receptor signaling is required for the Th17-inducing effect of CAF01 *in vivo* (62). Here, we found that Etanercept strongly impaired the expression of IL-6 and IL-1β in monocytes stimulated with the Mincle ligand TDB, complementing the *in vivo* requirement for TNFR1 on myeloid cells for Th17 generation and supporting the interpretation that TNFR1 enables monocytes and macrophages to provide signal 3. Together, the results from using LysM-Cre, Clec9a-Cre and CD11c-Cre deleter mice suggest that antigen presentation by cDC is independent from TNFR1 signaling, whereas signal 3 is derived mainly from monocytes/macrophages, activated by the Mincle-dependent adjuvant if TNFR1 is functional.

The specificity of Cre-mediated TNFR1 deletion in different immune cells was overall according to our expectations, but it is difficult to target with the used Cre deleters only one specific cell type. In relation to our results, we notice that the phenotype observed after LysM-Cre-mediated deletion of TNFR1 may originate beside from its signaling in monocytes also from macrophages or another unidentified population. We found TNFR1 expression only in a small subset of macrophages, making them an unlikely cell population to explain the TNF-dependent CAF01 adjuvanticity. Already the initial paper reporting LysM-Cre deleter mice reported the targeting of some CD11c+ DC (44). By studying TNFR1 expression and deletion on cDC1 and cDC2, we limited our analyses to the most abundant DC populations. It would be interesting to test TNFR1 expression in other DC subsets, especially to what extent TNFR1 on inflammatory DC will be targeted by the CD11c-Cre-mediated deletion. It was shown that myeloid-derived inflammatory DC promote human T cell differentiation towards Th17 (63). This could be an alternative explanation for the observed partial impairment of the vaccine response by the CD11c-Cre-mediated TNFR1 deletion. Additionally, we cannot exclude that LysM-driven Cre deletes the TNFR1 on inflammatory DC. The distinction between monocytes, macrophages and different DC subsets using the existing markers is not unequivocal and subject to adjustments as new insights are published. Very recently, the description of the DC3 lineage by Ginhoux and colleagues (64) as Th17-inducing cells derived from Ly6C^+^ monocyte-DC precursors is of special interest because fate mapping in LysM-Cre-R26TdT mice showed a high proportion of marked DC3. It is therefore possible that the myeloid cell populations required for Th17 induction, which we considered as monocytes/macrophages, may also contain DC3. On the other hand, CD11c-Cre shows leaky expression in different hematopoietic lineages, which can lead to deletion of sensitive floxed alleles (65). With regard to CD11c-Cre, we observed a partial deletion of TNFR1 on macrophages and Ly6C^low^ monocytes, which may reduce cytokine release and thereby inhibit Th17 generation, rather than the intended deletion of TNFR1 in cDCs.

Based on reports showing that TNF block can reduce Th17 and Th1 cell differentiation of naïve human T cells *in vitro* (66, 67), we considered that TNF may act directly on CD4^+^ T cells to promote Th17 differentiation and therefore included the Lck-Cre deleter strain. TNFR1 surface protein levels were relatively modest in T cells and we found no evidence for an important role of the TNFR1 on T cells in Th17 induction. However, we note that the effects of TNF on T cells may depend on signaling through TNFR2, which we did not address here. First, TNF signaling via TNFR2, but not TNFR1, acts as a costimulatory signal for T cell receptor activation in human T cells (68). In addition, a recent publication revealed that TNF signaling through TNFR2, together with TGF-β, enhances Th17 induction (69). Clearly, the potential involvement of TNFR2 in regulating vaccination-induced Th cell differentiation deserves further investigation.

Besides the direct effects on T cells, it was shown in rheumatoid arthritis (RA) patients that TNF induces IL-6 in monocytes, via TNFR1 and TNFR2, which leads to an increase in Th17 cells (70). This reflects our finding that Etanercept prevents the induction of IL-6 in murine monocytes. TNF can also promote differentiation of human monocytes into DC capable of inducing Th17 responses (71). Alternatively, an indirect effect was described in RA patients treated with the TNF blocker Adalimumab, where regulatory T cells increase and inhibit IL-6 production in monocytes via IL-10 (72). We did not find increased IL-10 levels in mice after TNF blockage by Etanercept, arguing that this mechanism may be specific to RA or humans, or that the type of TNF blocker makes a difference.

We also analyzed whether TNF/TNFR1 signaling contributes to the generation of specific antibodies after H1/CAF01 immunization. Knockout of TNF in mice prevents germinal center (GC) formation, disrupts follicular DC networks and consequently leads to impaired antibody responses (73). Even transient blockade of TNF by Etanercept can have an adverse effect on GC organization and reduce the number of follicular DCs (74). TNFR1 contributes to GC formation through its role in follicular DC differentiation (75–77). In the H1/CAF01 immunization model, induction of specific antibodies was markedly reduced in whole body TNF knockout mice and blockade of TNF was moderately inhibitory. In contrast, TNFR1 deletion in myeloid cells and in DC did not affect antibody titers. Thus, the loss of antigen-specific Th17 cells observed in the myeloid TNFR1 knockout mice was not linked to antibody responses, indicating that, although Th17 cells can act *in vitro* as B cell helpers and drive an isotype switch towards IgG2a and IgG3 (78), the IgG2a response to H1/CAF01 is likely driven by Th1 cells (79).

We previously linked the requirement of TNF for the induction of an IL-17 response after immunization with the Mincle-dependent adjuvant CAF01 to upregulation of this PRR (34). We have now found that TNFR1 signaling on myeloid cells is essential for Th17 induction but dispensable for Th1 or humoral responses. We therefore postulate that the missing upregulation of Mincle after binding the adjuvant interrupts a positive TNF-dependent feed-forward loop, thwarting myeloid cell activation and secretion of Th17-polarizing cytokines. In line with our model, we found that after stimulation of monocytes with the CAF01 component TDB, the increase in Mincle expression was prevented by blocking TNF, accompanied by strongly reduced expression of IL-6 and IL-1β. Significantly, constitutive transgenic Mincle expression prevented the loss of the specific Th17 response caused by the TNF blocker, further supporting the notion that disrupted upregulation of Mincle in myeloid cells impairs the sensing of the TDB adjuvant. The transgenic Mincle expression did not fully protect Th17 induction from TNF blockade, indicating that TNF exerts additional effects beyond Mincle upregulation. Mincle-independent effects of TNF may act on myeloid cells through promotion of Th17 inducing cytokines, or directly on T cells, e.g. via TNFR2, and remain to be studied. The Mincle-transgenic mouse line was used before to study the role of Mincle signaling in pneumonia caused by pneumococci and by *Staphylococcus aureus* (80, 81). The ubiquitous expression of transgenic Mincle mRNA contrasts the specific expression of endogenous Mincle in myeloid cells. However, surface localization of Mincle protein requires the adapter protein Fc receptor gamma chain (encoded by *Fcer1g*) that interacts with the transmembrane domain of Mincle (82). Fc receptor gamma expression is restricted to myeloid cells, mast cells, and innate lymphoid cells, including NK and NKT cells. Indeed, in addition to myeloid cells and some DC, we found robust transgenic Mincle protein expression on NK1.1^+^ cells; however, these cells did not contribute to Th17 induction, as shown by antibody-mediated depletion experiments.

In summary, we found that Mincle upregulation is a mechanism through which TNF enhances adjuvant recognition and responsiveness. This process is crucial in myeloid cells, especially in monocytes, where the lack of TNFR1 signaling prevents the secretion of Th17-promoting cytokines. It will be of interest to determine the contribution of TNFR1 signaling to Th17 induction by other vaccine adjuvants. Th17 induction by the widely used, experimental Complete Freund’s Adjuvant containing heat-killed *Mycobacterium tuberculosis* is dependent on Mincle and its signaling molecule Card9, as is the expression of IL-6, IL-1β, and IL-23 at the injection site (27). Thus, given its dependence on Mincle, we consider it likely that Complete Freund’s Adjuvant requires TNFR1 signaling in myeloid cells to generate a strong Th17 response. Similarly, TNF-regulated Mincle likely contributes to the induction of Th17 by BCG or by recombinant MTB protein vaccines adjuvanted with DDA plus TDM and MPL observed and dissected in a series of papers by the Khader group (20, 21). On the other hand, Mincle expression is induced by the TLR4 ligand LPS in a TNF-independent manner and the synthetic TLR4-activating adjuvant G3D6A generates Th17 cells in Etanercept-treated mice (34). Therefore, the abrogation of Th17 induction by Mincle-dependent adjuvants may be overcome by including a TLR-activating ligand. Recently, combination adjuvants stimulating both Mincle and TLR9 have been developed and shown superior activity (83). Similarly, the coadministration of CAF01 with the live BCG vaccine has remarkably enhanced Th17 cell induction and vaccine protective efficacy (84). It will be important to examine whether the provision of combined stimulation may render Th17 induction resistant to TNF blockade. If so, such adjuvant cocktail vaccines would be promising for immunization of patients receiving treatment for inflammatory disease with TNF-blocking biologicals.

Mincle acts as receptor not only for the mycobacterial cord factor, but also binds a considerable number of microbial glycolipids, as well as endogenous damage-associated molecular patterns. Therefore, Mincle and its regulation by TNF on myeloid cells could also be relevant for other, clinically used vaccines. The widely used aluminum salt-based adjuvants stimulate the immune system through uric acid and DNA released during cell death (85, 86). Mincle binds several molecules released by dying cells, such as spliceosome-associated protein 130 (SAP130) (82) and β-glucosylceramide (87). Thus, Mincle may contribute to the immunogenicity of the aluminum adjuvant by recognizing additional released DAMPs. Among pathogens, the pneumococcal cell wall glycolipid glucosyl-diacylglycerol (Glc-DAG) binds and activates Mincle (81, 88). Whether Glc-DAG is contained in pneumococcal polysaccharide vaccine preparations and acts as a built-in Mincle-dependent adjuvant is yet unknown. If so, the reduced immunogenicity of the pneumococcal polysaccharide vaccine (9, 10) in patients receiving TNF blockers could be caused by a similar mechanism. Future studies should also address the role of Mincle in the adverse effects of TNF blockade on different vaccine adjuvants. Molecularly defined adjuvant molecules specifically targeting TLRs, CLRs or cytosolic PRRs have propelled the development of efficient subunit vaccines directing distinctive Th cell and antibody responses (22). Dissecting the orchestration of adaptive immunity by the interplay of cytokine generation and signaling in different target cells in response to adjuvants, as presented here for CAF01, Mincle and TNF, provides valuable knowledge to further improve the design of modern vaccines and to optimize their application.

## Data Availability

The original contributions presented in the study are included in the article/**Supplementary Material**. Further inquiries can be directed to the corresponding author.
