# Supplementary material for: Mincle-dependent Th17 adjuvanticity requires TNFR1 signaling in myeloid cells
Source: Front Immunol. 2026 Feb 9;17:1746618. doi: 10.3389/fimmu.2026.1746618 (PMC12926142; doi:10.3389/fimmu.2026.1746618)
Supplement: Supplementary file 1 [file DataSheet1.docx]

Supplementary Material

| **Table S1: antibodies used in flow cytometry** | | | | | | | |
| --- | --- | --- | --- | --- | --- | --- | --- |
| **target** | **host species** | **isotype** | **fluorophore** | **dilution**  **1:x** | **clone** | **producer** | **catalog number** |
| CD11b | Rat | IgG2b, κ | FITC | 400 | M1/70 | BioLegend | 101206 |
| CD11b | Rat | IgG2b, κ | BV510 | 400 | M1/70 | BioLegend | 101245 |
| CD11c | Armenian Hamster | IgG | PE-Cy7 | 200 | N418 | BioLegend | 117318 |
| CD11c | Armenian Hamster | IgG | PerCp Cy5.5 | 100 | N418 | BioLegend | 117327 |
| CD11c | Armenian Hamster | IgG | BV421 | 400 | N418 | BioLegend | 117329 |
| CD19 | Rat | IgG2a, κ | BV421 | 100 | 6D5 | BioLegend | 115549 |
| CD19 | Rat | IgG2a, κ | APC eF780 | 400 | eBio1D3 | invitrogen | 47-0193-82 |
| CD19 | Rat | IgG2a, κ | FITC | 100 | 6D5 | BioLegend | 115505 |
| CD19 | Rat | IgG2a, κ | PE | 50 | 6D5 | BioLegend | 115507 |
| CD26 | Rat | IgG2a, κ | FITC | 100 | H194-112 | BioLegend | 137806 |
| CD3 | Armenian Hamster | IgG | BV421 | 100 | 145-2C11 | BioLegend | 100335 |
| CD3 | Armenian Hamster | IgG | FITC | 100 | 145-2C11 | BioLegend | 100305 |
| CD3 | Armenian Hamster | IgG | APC eF780 | 400 | 145-2C11 | invitrogen | 47-0031-82 |
| CD3 | Armenian Hamster | IgG | BUV 737 | 100 | 145-2C11 | BD | 612771 |
| CD4 | Rat | IgG2b, κ | FITC | 400 | GK1.5 | BioLegend | 100405 |
| CD64 | Mouse | IgG1, κ | PE-Cy7 | 100 | X54-5/7.1 | BioLegend | 139314 |
| CD8 | Rat | IgG2a, κ | APC eF780 | 100 | 53-6.7 | BioLegend | 100711 |
| CD8 | Rat | IgG2a, kappa | eF450 | 400 | 53-6.7 | eBioscience | 48-0081-82 |
| CD88 | Rat | IgG2b, κ | APC | 100 | 20/70 | BioLegend | 135807 |
| F4/80 | Rat | IgG2a, κ | APC | 100 | BM8 | BioLegend | 123115 |
| F4/80 | Rat | IgG2a, κ | PE | 200 | BM8 | BioLegend | 123109 |
| gd TCR | Armenian Hamster | IgG | APC | 100 | GL3 | BioLegend | 118134 |
| IFNg | Rat | IgG1, κ | APC | 400 | XMG1.2 | eBioscience | 17-7311-82 |
| IL10 | Rat | IgG2b | PE | 100 | JES5-16E3 | BD | 561060 |
| IL17 | Rat | IgG2a, κ | PE-Cy7 | 100 | eBio17B7 | invitrogen | 25-7177-82 |
| Isotype control | Rat | IgG1 kappa | unconjugated | 200 | eBRG1 | invitrogen | 14-4301-82 |
| isotype control | Armenian Hamster | IgG | PE | 100 | HTK888 | BioLegend | 400907 |
| Ly6C | Rat | IgG2c, κ | perCP-Cy5.5 | 200 | HK1.4 | BioLegend | 128011 |
| Ly6G | Rat | IgG2a, κ | APC-Cy7 | 100 | 1A8 | BioLegend | 127624 |
| Ly6G | Rat | IgG2a, κ | PE-Cy7 | 400 | 1A8 | BioLegend | 127617 |
| MHC II (I:A/I:E) | Rat | IgG2a, κ | BUV395 | 400 | 2G9 | BD | 740295 |
| Mincle | Rat | IgG2a | unconjugated | 200 | 4A9 | MBL | D292-3 |
| NK1.1 | Mouse | IgG2a | BUV395 | 100 | PK136 | BD | 740771 |
| NK1.1 | Mouse | IgG2a | APC eF780 | 100 | PK136 | invitrogen | 47-5941-82 |
| NK1.1 | Mouse | IgG2a | PE-Cy7 | 100 | PK136 | eBioscience | 25-5941-82 |
| NK1.1 | Mouse | IgG2a | FITC | 100 | PK136 | eBioscience | 25-5941-82 |
| NKp46 | Rat | IgG2a, κ | PE | 100 | 29A1.4 | BioLegend | 137602 |
| rat IgG | mouse | IgG1 | APC | 100 | R1-12D10 | invitrogen | 17-4812-82 |
| TNFR1 | Rat | IgG1 | PE | 100 | 55R-286 | BioLegend | 113902 |
| XCR1 | Rat | IgG2a, κ | BV421 | 400 | ZET | BioLegend | 148212 |

| **Table S2: for the qPCR used primer and probes** | | | | |
| --- | --- | --- | --- | --- |
| **gene** | **primer forw** | **primer seq rev** | **probe** | **supplier** |
| *Hprt* | cttacctcactgctttccgg | catcatcgctaatcacgacg | (6FAM) ttttgccgcgagccgaccggtcc (OQA) | Sigma-Aldrich |
| *Mincle* | accagatgtgtcgtaaca | tccagttcaaaggacagc | (6FAM) ttgactgaacctgatgcctcact (BHQ1) | Sigma-Aldrich |
| *IL6* | gctaccaaactggatataatcagga | ccaggtagctatggtactccagaa | universal probe 6 | Roche |
| *Il1b* | ttgacggaccccaaaagat | agctggatgctctcatcagg | universal probe 38 | Roche |


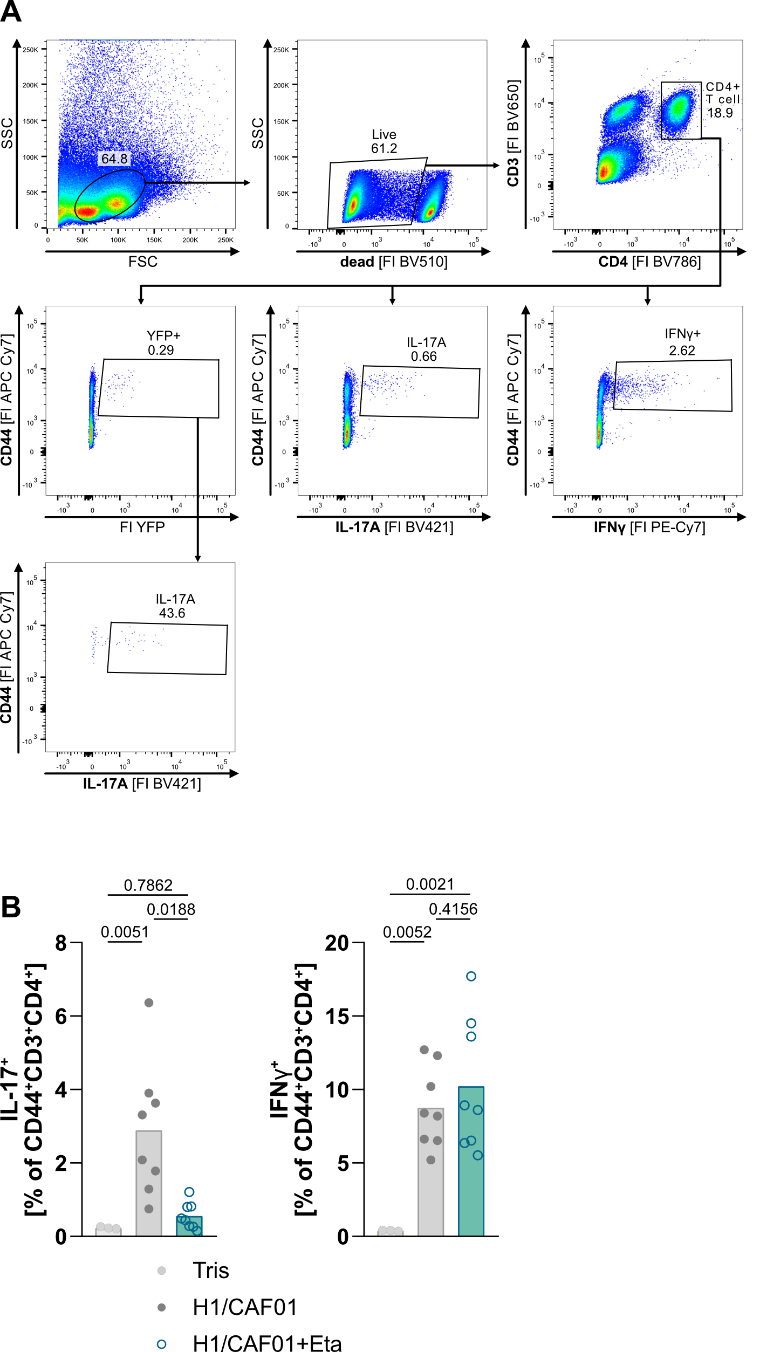


**Supplementary Figure 1: Measurement of Th17 cells after CAF01 immunization** Spleen cells from control or immunized IL-17A fate reporter mice were prepared and stained. Singlet live CD4^+^ T cells were gated as shown (A) to determine the number of CD3^+^ CD4^+^ cells expressing YFP as a result of Il17a-Cre activity, or producing IL-17 or IFNγ after specific restimulation with H1 by intracellular staining. The same cell populations from Fig. 1 were additionally displayed as a percentage of CD44^+^, CD4^+^, CD3^+^ cells (B).

**
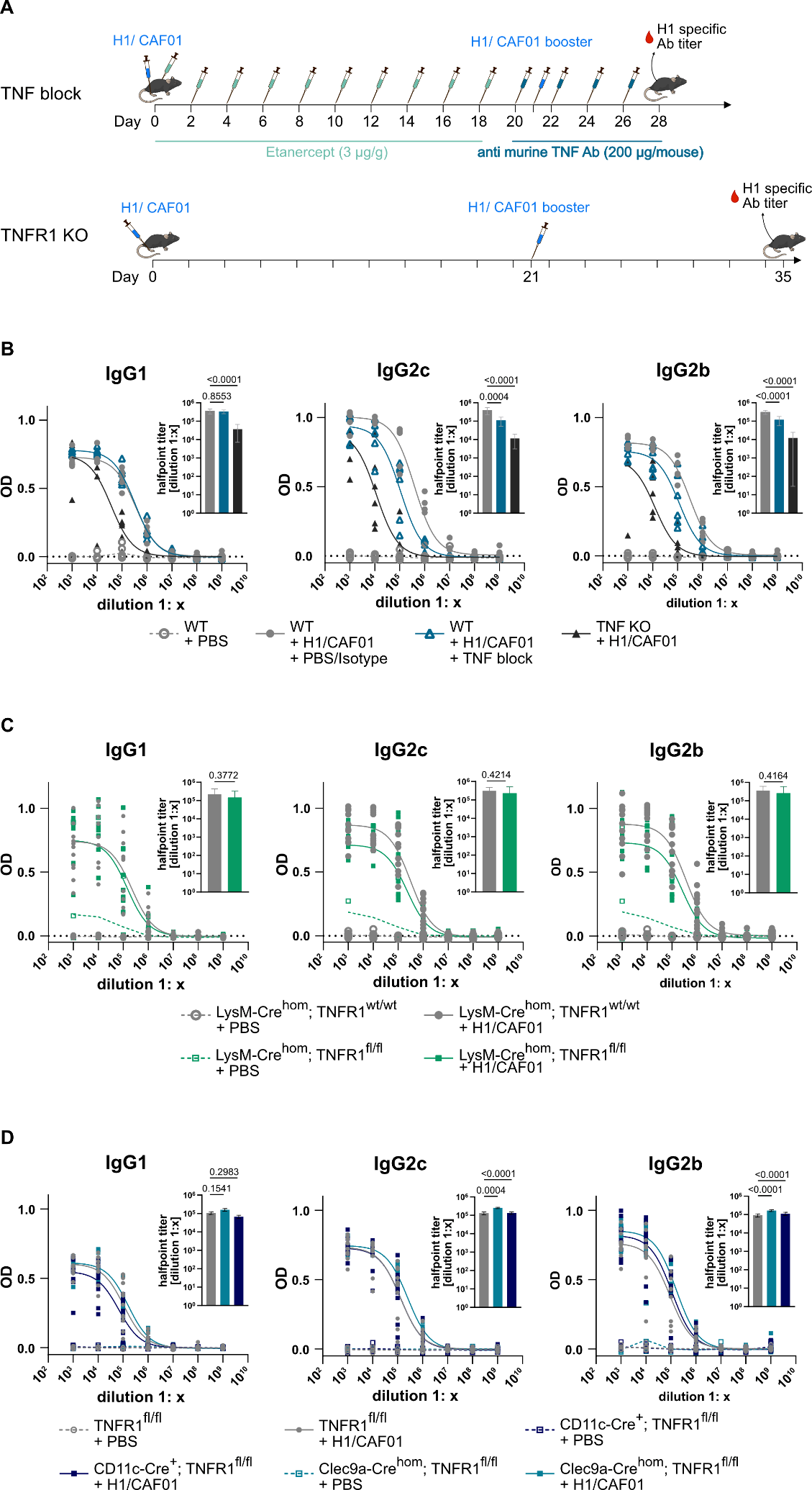
**

**Supplementary Figure 2.** **TNF is necessary to generate H1-specific antibodies, but the TNFR1 on myeloid cells or DC is dispensable**. Mice were immunized twice with H1/CAF01, and H1-specific Ab titers were determined in the sera by ELISA (A). Ab titers were compared between immunized WT mice, WT mice immunized in the presence of TNF block, and immunized TNF^-/-^ mice (B). LysM-Cre^hom^; TNFR1^fl/fl^ mice were compared to LysM-Cre^hom^; TNFR1^wt/wt^ mice (C). CD11c-Cre^+^; TNFR1^fl/fl^ and Clec9a-Cre^hom^; TNFR1^fl/fl^ mice were compared to TNFR1^fl/fl^ controls (D). For the immunized groups (n = 6 – 12 mice per group), the correlation between the measured OD values and the respective dilution step was modeled using three-parameter non-linear regression. From this, the midpoint titers were calculated (displayed as mean with SD), and differences were tested by one-way ANOVA followed by Dunnett’s multiple comparison test (B, D) or Student's t-test (C).

**
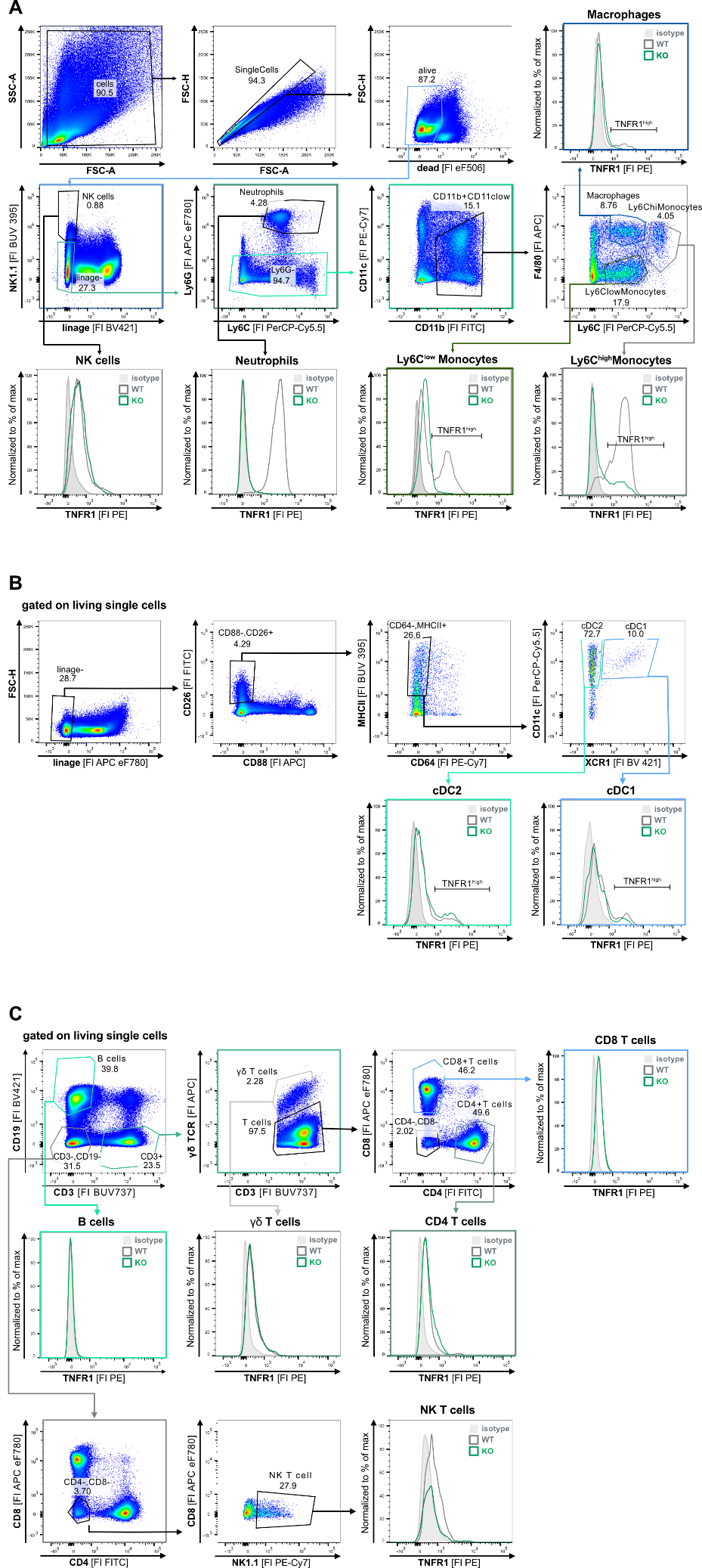
**

**Supplementary Figure 3.** **Gating strategy for TNFR1 quantification on splenocytes.** To determine TNFR1 expression via flow cytometry, three different subpanels were used. The gating strategy to determine myeloid cells (A), DC subsets (B), and lymphocytes (C) is demonstrated for one representative sample. Overlay histogram of LysM-Cre^hom^; TNFR1^wt/wt^ (gray line), LysM-Cre^hom^; TNFR1^fl/fl^ (green line), and isotype control (light gray filled graph). For some cell populations, a bimodal expression of the TNFR1 was observed; for these cells, an additional gate was used to define the TNFR1high cells.


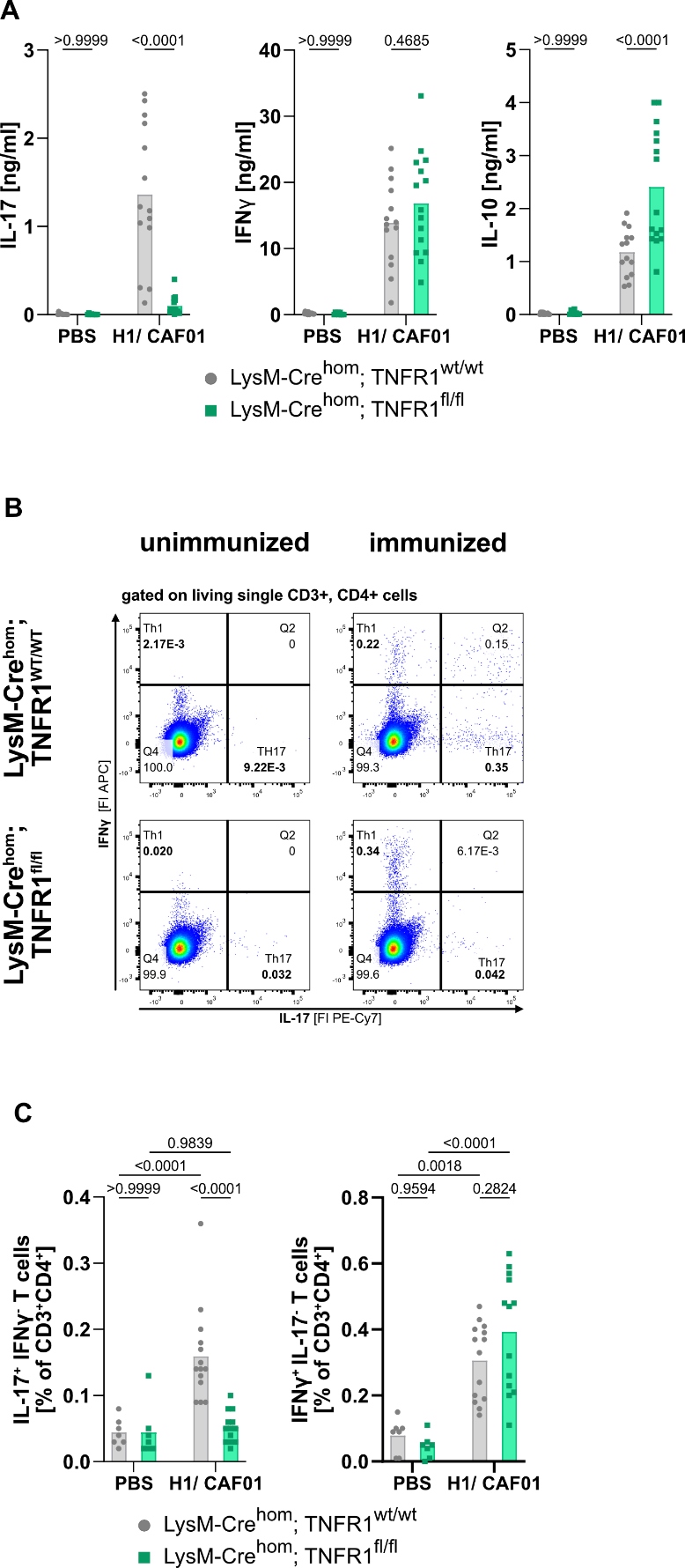


**Supplementary Figure 4.** **The deletion of the TNFR1 on myeloid cells prevents the induction of the specific Th17 response in the spleen 7 days after H1/CAF01 immunization, comparable to the results obtained from the draining LN.** LysM-Cre^hom^; TNFR1^fl/fl^ and LysM-Cre^hom^; TNFR1^wt/wt^ mice were immunized s.c. in the footpad with H1/CAF01. The mice were killed 7 days after immunization. Cells from the spleen were isolated, and secreted cytokines were measured via ELISA 4 days after restimulation with H1 protein (A). The amount of IL-17 or IFNγ-producing CD3^+^ CD4^+^ cells in the spleen was measured after H1 peptide restimulation using flow cytometry. A representative plot of the intracellular cytokine staining (B) and the respective quantification, pooled from three experiments (C). Pooled from three independent experiments (n = 7-14 mice per group in total), two-way ANOVA was followed by Sidak’s multiple comparison test (A, C).

**
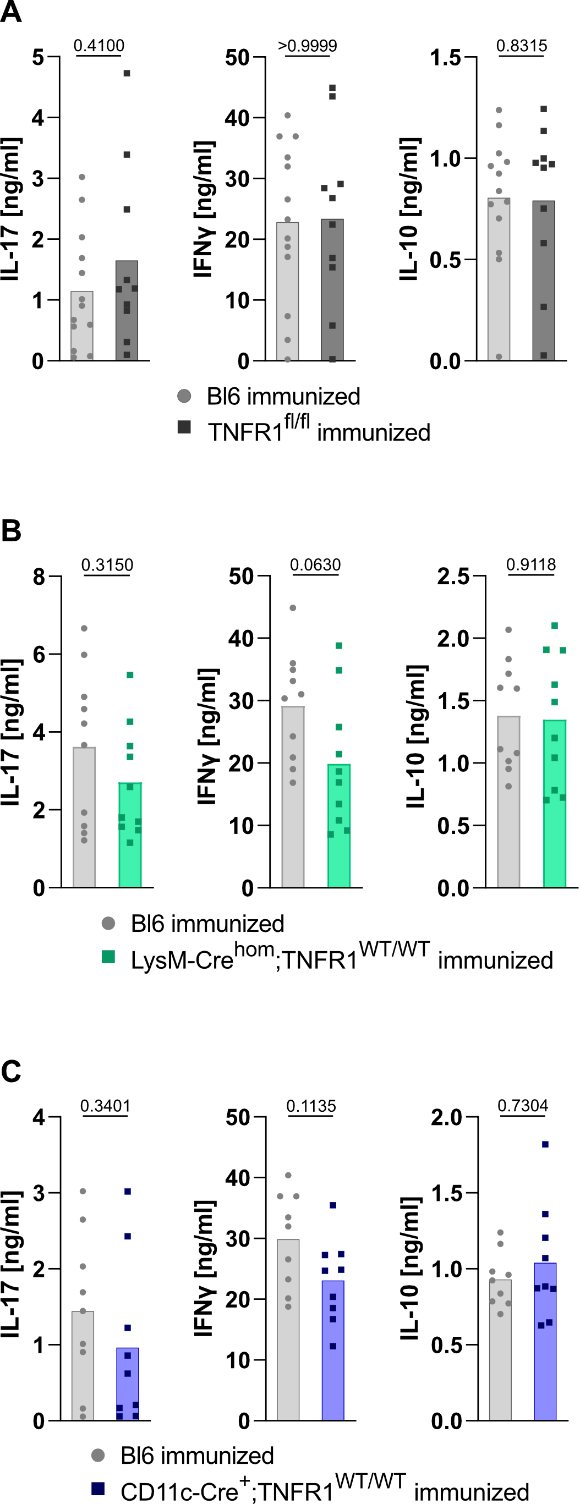
**

**Supplementary Figure 5.** **Floxing of the TNFR1 gene or insertion of the Cre recombinase alone do not impair the IL-17 response.** The influence of the floxed TNFR1 exons (A), as well as the effect of the Cre recombinase knock-in in the LysM locus (B) and the Cre expression in the CD11c-Cre^+^ mice (C), on the immunization was tested. To this end, the specific Cytokine production was measured by ELISA after restimulation of LN cells of immunized mice (n = 9 – 13 mice per group). Mann-Whitney U test (A, B, C).


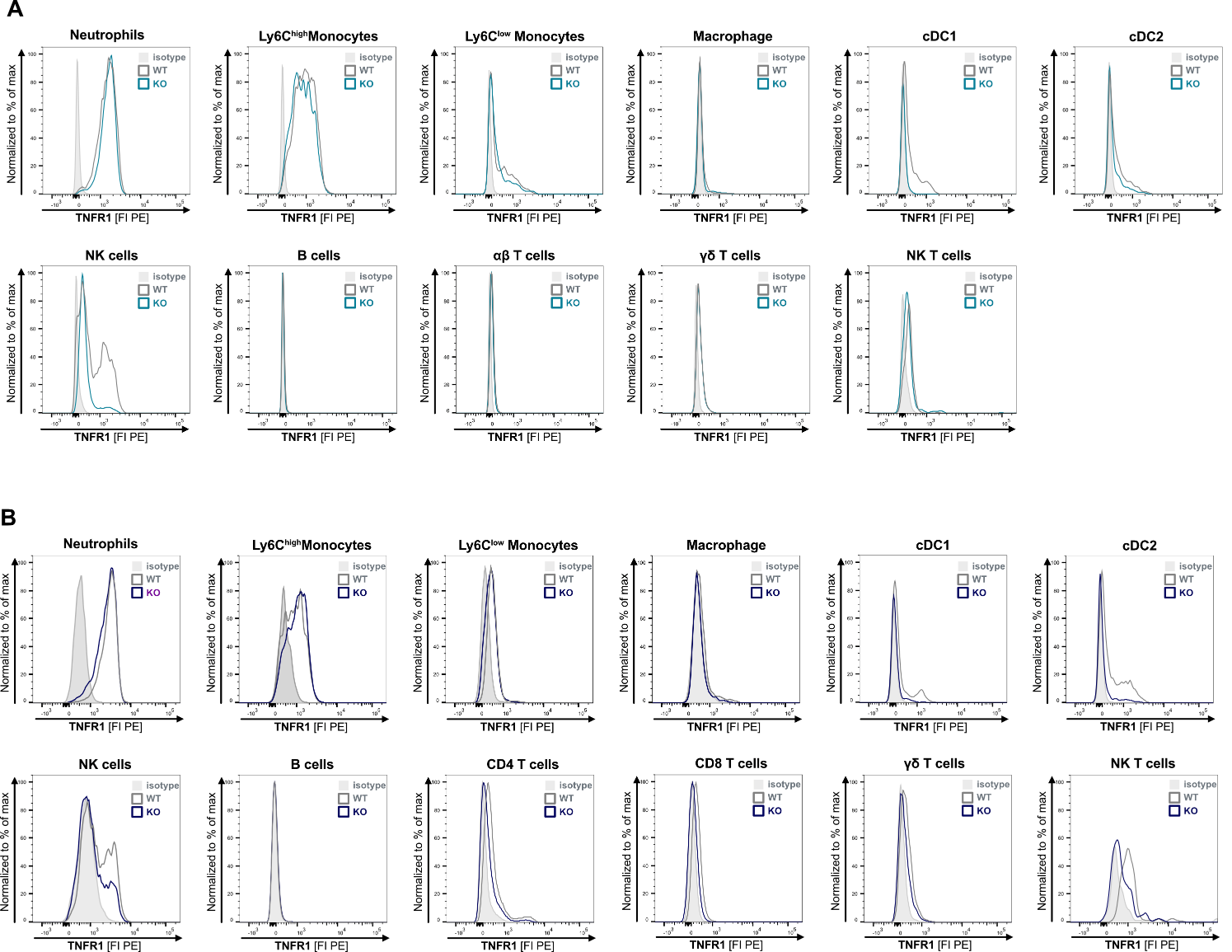


**Supplementary Figure 6.** **TNFR1 expression on different cell types after Clec9a-Cre- and CD11c-Cre-mediated deletion.** The TNFR1 expression on different cell types from spleen was measured via flow cytometry staining in Clec9a-Cre^het^; TNFR1^fl/fl^ (A) and CD11c-Cre^+^; TNFR1^fl/fl^ mice (B) and compared to control mice. One representative example is displayed. TNFR1^fl/fl^ is displayed in the histogram with a gray line for A and B. The TNFR1 expression on Clec9a-Cre^het^; TNFR1^fl/fl^ (A) and the CD11c-Cre^+^; TNFR1^fl/fl^ (B) splenocytes with a light blue or dark blue line, respectively. The isotype control is illustrated with a light gray-filled graph. The gating strategy is similar to what was shown before (Fig. S3 A-C)


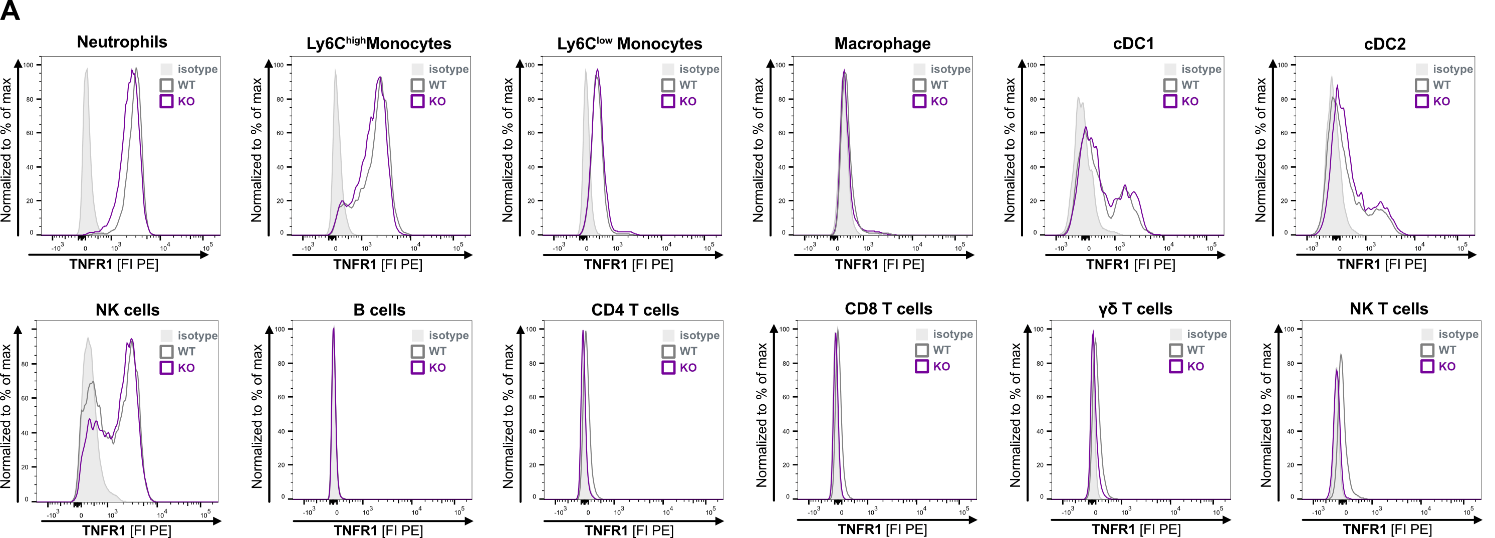


**Supplementary Figure 7.** **TNFR1 expression on different cell types after Lck-Cre-mediated deletion.** The TNFR1 expression on different spleen cell types was measured via flow cytometry staining in Lck-Cre^+^; TNFR1^fl/fl^ mice and compared to TNFR1^fl/fl^ control mice. One representative example is displayed (A). The TNFR1^fl/fl^ is displayed in the histogram with a gray line. The Lck-Cre^+^; TNFR1^fl/fl^ with a purple line. The isotype control is illustrated with a light gray-filled graph. The gating strategy is similar to what was shown before (Fig. S3 A-C).


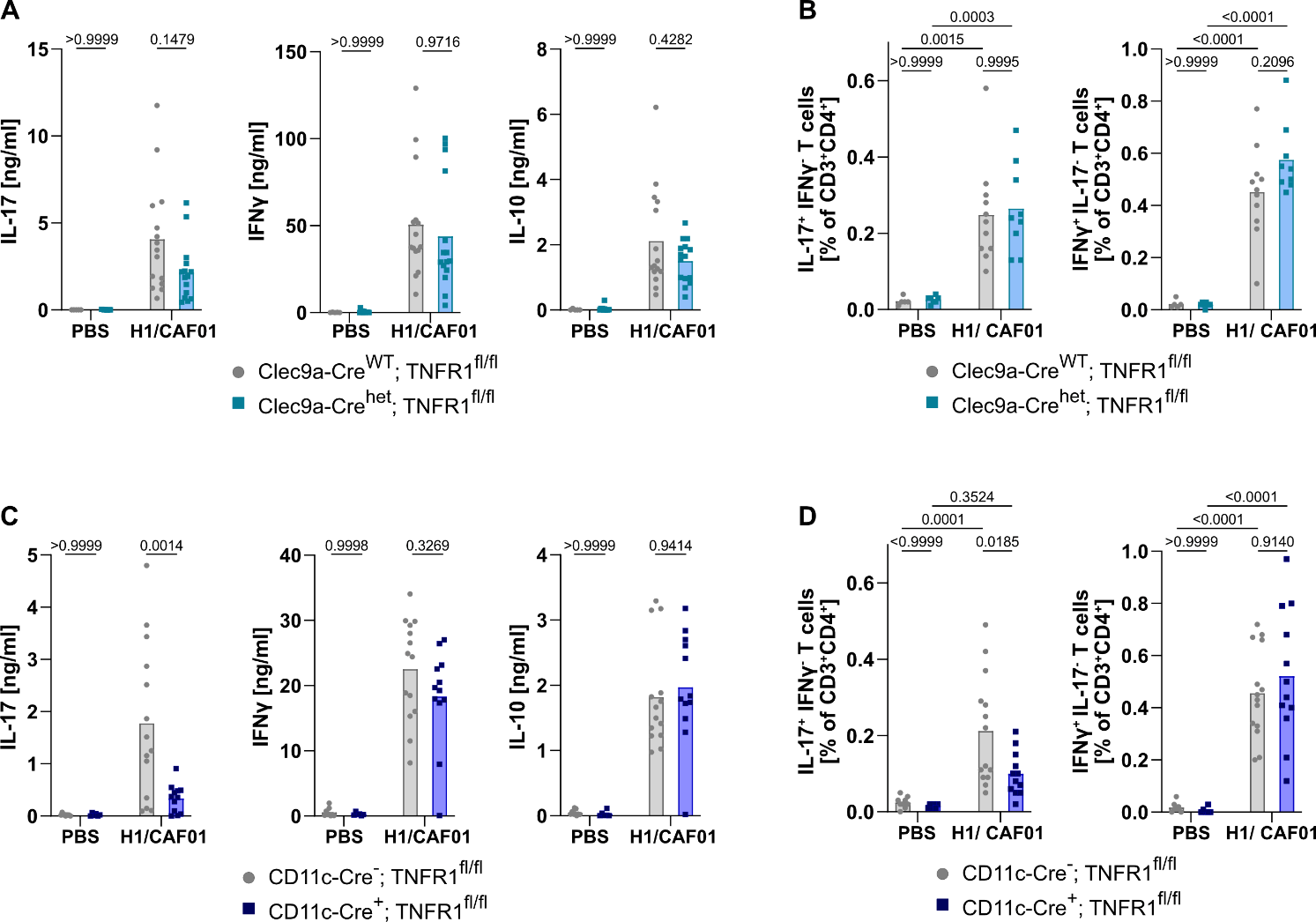


**Supplementary Figure 8.** **Additional spleen data confirm that the specific Th17 response after H1/CAF01 immunization does not depend completely on the TNFR1 on DC.** Clec9a-Cre^het^; TNFR1^fl/fl^ and CD11c-Cre^+^; TNFR1^fl/fl^ mice as two different DC deletion models and TNFR1^fl/fl^ mice as controls were used. The mice were immunized s.c. in the footpad with H1/CAF01 and killed 7 days later. Splenocytes were isolated, and secreted cytokines were measured via ELISA 4d after restimulation with H1 protein for the Clec9a-Cre (A) and CD11c-Cre (C) deletion models. The amount of IFNγ or IL-17-producing Th cells in the spleen after H1 peptide restimulation was measured using flow cytometry for Clec9a-Cre (B) and CD11c-Cre (D) mediated deletion. Pooled from three independent experiments (n = 6-14 mice per group in total), two-way ANOVA, followed by Sidak’s multiple comparison test.


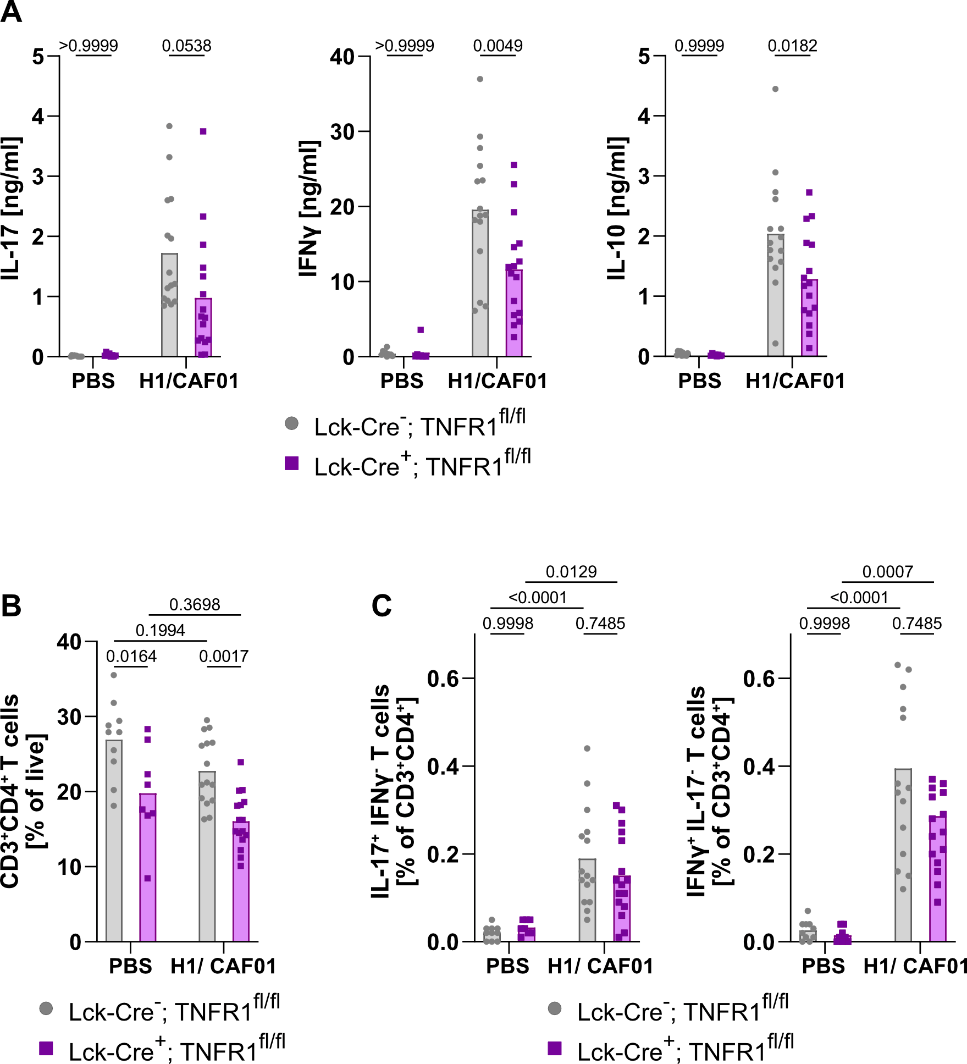


**Supplementary Figure 9.** **Additional spleen data confirm that the lack of the TNFR1 on T cells reduces IFNγ and IL-10, but does not impact the specific Th17 response in particular.** Lck-Cre^+^; TNFR1^fl/fl^ and TNFR1^fl/fl^ mice were immunized s.c. in the footpad with H1/CAF01 and killed 7 days later. Splenocytes were isolated, and secreted cytokines were measured via ELISA 4 days after restimulation with H1 protein (A). The frequency of Th cells (CD3^+^ CD4^+^) in the spleen was measured via flow cytometry (B). The amount of IFNγ and IL-17-producing Th cells in the spleen after H1 peptide restimulation was measured using flow cytometry (C). Pooled from three independent experiments (n = 8-16 mice per group in total), two-way ANOVA, followed by Sidak’s multiple comparison test.


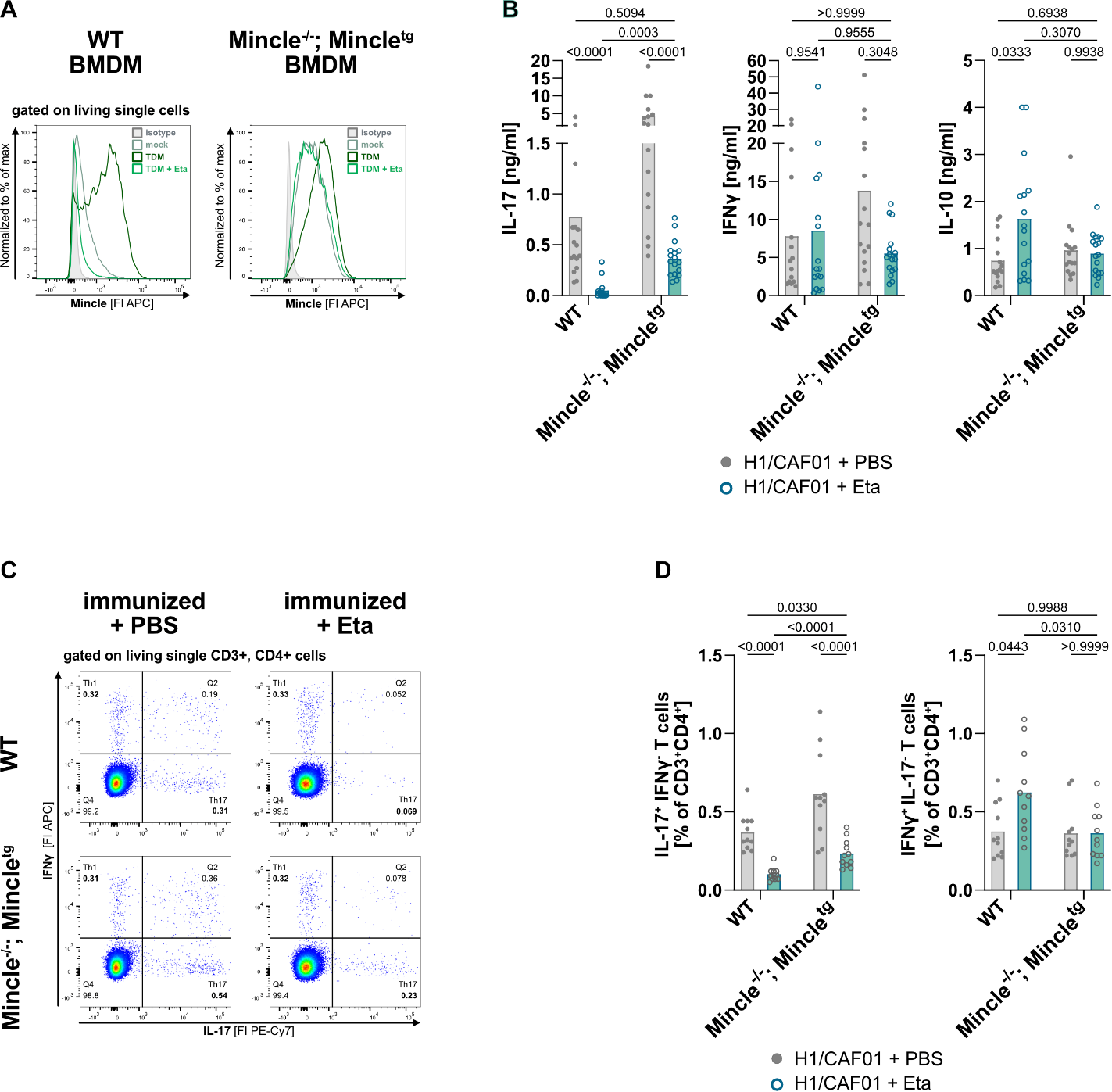


**Supplementary Figure 10.** **The impairment of the Th17 response by the TNF blocker Etanercept can be prevented by constitutive Mincle expression, also in the spleen.** The Mincle expression on WT and Mincle^-/-^; Mincle^tg^ BMDM was measured after TDM stimulation, in the presence of the TNF blocker Etanercept (Eta), via flow cytometry. One representative sample is displayed (A). WT and Mincle^-/-^; Mincle^tg^ mice were immunized with H1/CAF01 in the presence or absence of the TNF blocker Etanercept (Eta). The mice were killed after 7 days (similar to Fig. 7B). Cells from the spleen were isolated, and secreted cytokines were measured via ELISA 4 days after restimulation with H1 protein (B). The amount of IL-17 or IFNγ-producing Th cells in the spleen was measured after H1 peptide restimulation using flow cytometry (C and D). Pooled from three independent Experiments (n = 8-16 mice per group in total), two-way ANOVA followed by Sidak’s multiple comparison test performed on log-transformed data (B, D)


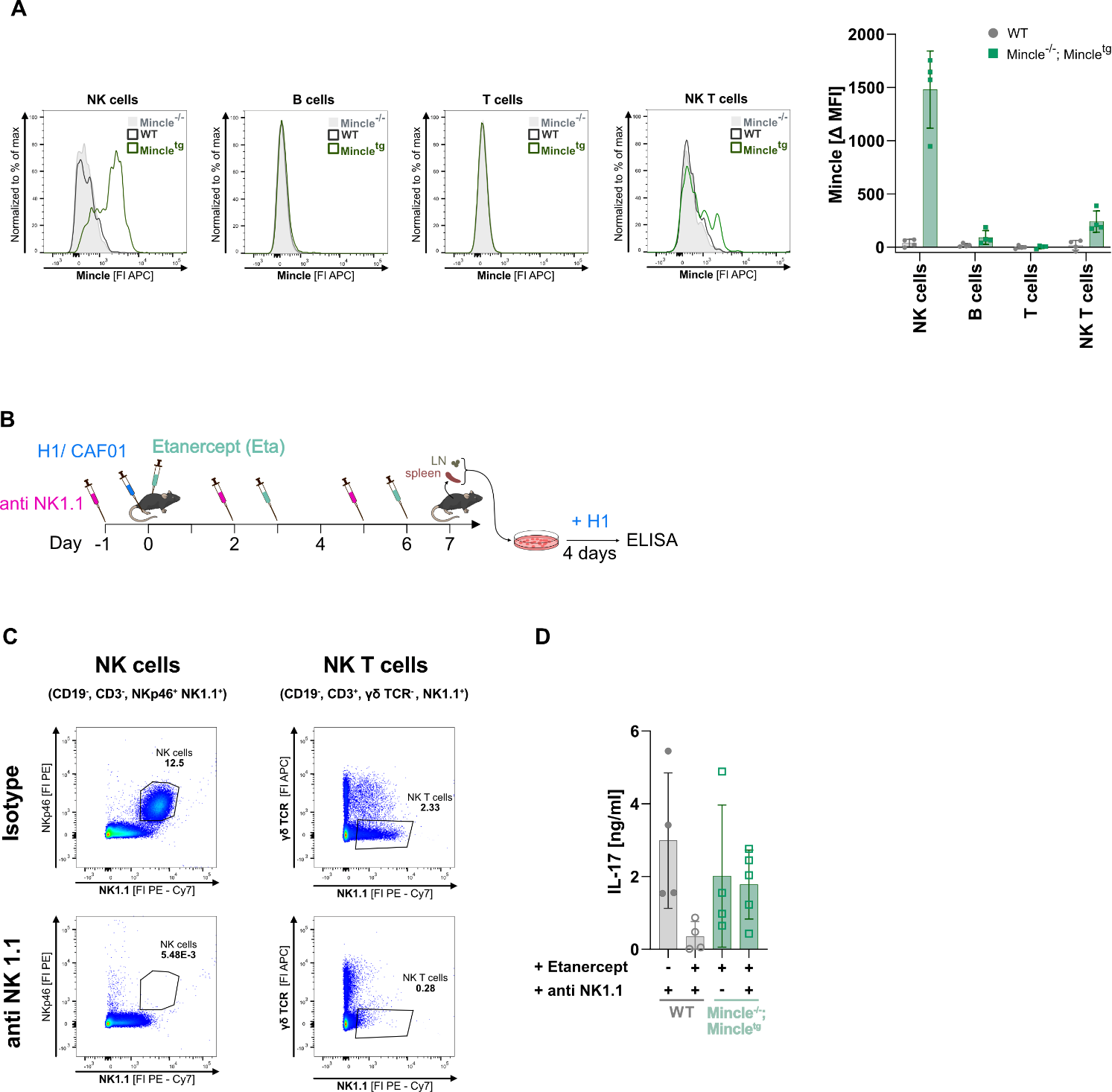


**Supplementary Figure 11.** **NK1.1-positive cells are dispensable for IL-17 production and rescue in the mincle transgenic mice.** The Mincle expression was measured on spleen cells isolated from unimmunized WT (gray line) and Mincle^-/-^; Mincle^tg^ mice (green line) by flow cytometry, staining obtained from Mincle KO mice (filled gray histogram) was used for the quantification of the ΔMFI (A, the populations were defined similarly to Fig. S3A, C). WT and Mincle^-/-^; Mincle^tg^ mice were immunized with H1/CAF01 in the presence or absence of the TNF blocker Etanercept. Additionally, NK1.1-positive cells were depleted using an anti-NK1.1 antibody (250 µg/mouse; see B for experimental design). The successful depletion of NK1.1^+^ cells was checked on day 7 via flow cytometry; one representative example is displayed (C). Cells from the spleen were isolated, and secreted cytokines were measured via ELISA 4 days after restimulation with H1 protein (D). (Mean and SD of 4 mice per group)
